# MAPK Phosphatase AP2C3 Induces Ectopic Proliferation of Epidermal Cells Leading to Stomata Development in Arabidopsis

**DOI:** 10.1371/journal.pone.0015357

**Published:** 2010-12-23

**Authors:** Julija Umbrasaite, Alois Schweighofer, Vaiva Kazanaviciute, Zoltan Magyar, Zahra Ayatollahi, Verena Unterwurzacher, Chonnanit Choopayak, Justyna Boniecka, James A. H. Murray, Laszlo Bogre, Irute Meskiene

**Affiliations:** 1 Max F. Perutz Laboratories, Vienna Biocenter, University of Vienna, Vienna, Austria; 2 Max-Planck-Institute of Molecular Plant Physiology, Potsdam, Germany; 3 Institute of Biotechnology, University of Vilnius, Vilnius, Lithuania; 4 School of Biological Sciences, Royal Holloway University of London, Egham, United Kingdom; 5 Biological Research Centre, Institute of Plant Biology, Szeged, Hungary; 6 Cardiff School of Biosciences, Cardiff University, Cardiff, United Kingdom; Iowa State University, United States of America

## Abstract

In plant post-embryonic epidermis mitogen-activated protein kinase (MAPK) signaling promotes differentiation of pavement cells and inhibits initiation of stomata. Stomata are cells specialized to modulate gas exchange and water loss. Arabidopsis MAPKs MPK3 and MPK6 are at the core of the signaling cascade; however, it is not well understood how the activity of these pleiotropic MAPKs is constrained spatially so that pavement cell differentiation is promoted only outside the stomata lineage. Here we identified a PP2C-type phosphatase termed AP2C3 (Arabidopsis protein phosphatase 2C) that is expressed distinctively during stomata development as well as interacts and inactivates MPK3, MPK4 and MPK6. AP2C3 co-localizes with MAPKs within the nucleus and this localization depends on its N-terminal extension. We show that other closely related phosphatases AP2C2 and AP2C4 are also MAPK phosphatases acting on MPK6, but have a distinct expression pattern from AP2C3. In accordance with this, only AP2C3 ectopic expression is able to stimulate cell proliferation leading to excess stomata development. This function of AP2C3 relies on the domains required for MAPK docking and intracellular localization. Concomitantly, the constitutive and inducible AP2C3 expression deregulates E2F-RB pathway, promotes the abundance and activity of CDKA, as well as changes of CDKB1;1 forms. We suggest that AP2C3 downregulates the MAPK signaling activity to help maintain the balance between differentiation of stomata and pavement cells.

## Introduction

Signaling by mitogen activated protein kinases (MAPKs) regulates environmental responses and developmental processes in all eukaryotes. Signal-induced phosphorylation of MAPKs increases their activity [1], while dephosphorylation by protein phosphatases reverts the MAPK modules to an inactive state, signifying MAPK phosphatases as part of the mechanisms to ensure dynamics and tight regulation of signaling pathways.

In *Arabidopsis*, both environmental and inherent developmental signals, such as stomata development, are transmitted by the MAPKs MPK3 and MPK6, but how these very distinct signaling inputs generate specific responses is puzzling [2]. Stomata are two-cell structures surrounded by epidermal cells that allow the uptake of atmospheric carbon dioxide and release of oxygen and water vapor through the opening of the pore between stomatal guard cells. Environmental conditions, such as high light and CO_2_, affect stomata numbers in plants [3], pointing towards the cross talk between the environmental and developmental pathways. The cascade composed of the MAPKKK YODA, the MAPKKs MKK4/MKK5, and MAPKs MPK3/MPK6 promotes differentiation of epidermal cells and inhibition of stomatal initiation [4], [5]. The loss-of-function mutations or the absence of upstream signaling components, such as LRR receptor-like kinases (RLKs) or the receptor-like protein too many mouths (TMM) leads to abundance of stomata in clusters [6]. This signaling pathway likely responds to secreted peptide ligands such as the epidermis-intrinsic negative factors EPF1 and EPF2 that are produced by meristemoids, guard mother cells and young guard cells [7], [8], [9] and to STOMAGEN, a positive regulator of stomata density produced by mesophyll tissues [10]. How exactly the distribution of these ligands and the presence of signaling components in specific cell types set up the rules of spatial distribution of stomata and pavement cells within the epidermis is not fully understood.

The ERL-YODA-MAPK signaling pathway [4], [11], [12] activity is opposing the action of a group of related basic helix-loop-helix transcription factors that are successively ushering cells though the steps of stomata lineage: SPEECHLESS (SPCH) - initiation;, MUTE - proliferation; and FAMA - stomata differentiation [13], [14], [15], [16]. Phosphorylation of SPCH by MPK6 was shown to control the entry into stomata lineage and to connect signaling through MAPKs to downstream transcriptional regulatory targets of stomata development [13]. MAPKKs MKK4 and MKK5, as well as MKK7 and MKK9 inhibit the entry into stomata lineage and stomata proliferation by likely activating MPK3 and MPK6. On the other hand, MKK7 and MKK9 are able also to promote later stages of stomata differentiation possibly acting on MPK3/MPK6 or other MPKs [5]. This mode of action was suggested to set an inhibitory input at the entry point of the pathway, while for the cells that have already progressed well within stomata lineage to continue completing the stomata differentiation.

Protein phosphatases (PPs) counteract protein kinases by dephosphorylation, ensuring fast regulation of signaling. MAPK phosphatases control signaling pathways by their ability to dephosphorylate T - threonine and/or Y - tyrosine in the activation loop of MAPKs [17]. Thus, inactivation of MAPKs can be performed by different PPs, such as protein tyrosine phosphatases (PTPs), dual specificity phosphatases (DSPs) or protein phosphatases of type 2C (PP2Cs) [18], [19]. PTP and DSP phosphatases were shown to regulate MPK3/MPK6 pathways in plants [20], [21], [22], [23]. We found that PP2Cs AP2C1 and MP2C control stress-MAPKs in Arabidopsis and alfalfa, respectively [24], [25], [26]. ABI1 was shown to interact with MPK6 and to secure down-regulation of kinase activity [27]. These PP2Cs harbor in their protein structure a putative or confirmed kinase interaction motif (KIM). KIM has been found in many eukaryotic MAPK-interacting proteins, such as MAPKKs, transcription factors (TFs), PTPs, and demonstrated to be important for interaction with MAPKs [28]. This domain is present also in several members of cluster B Arabidopsis PP2C gene family: AP2C1, AP2C2, AP2C4 and AP2C3/AtPP2C5 [26], [29]. AP2C3 was previously classified as AtPP2C5 phosphatase [30], but its function and substrates remained unknown. Recently AP2C3/AtPP2C5 was shown to regulate stomata aperture, seed germination, abscisic acid-inducible gene expression and MAPK activation [31].We have demonstrated that AP2C1 controls MAPK activities, stress ethylene and plant innate immunity [26], however, it was unclear if the remaining KIM-containing PP2Cs from cluster B are MAPK phosphatases.

MAPKs are important regulators of cell proliferation in yeast and metazoans, however, in plants interaction between MAPK signaling and cell proliferation is little understood [32]. The duration of cell proliferation, the timing of the exit from proliferation to differentiation is thought to be controlled by an evolutionary conserved transcriptional regulatory switch, the E2F-RB pathway [33]. E2F and RB homologues have been identified in *Arabidopsis thaliana* [34] and their involvement in cell proliferation was demonstrated [35]. Similar to animal systems, plant E2Fs have been classified as transcriptional activators (E2FA and E2FB), or transcriptional repressor (E2FC) [35]. *Arabidopsis* retinoblastoma related protein (RBR1) could control the activities of these three E2Fs, but the developmental timing and role of RBR1 interaction with different E2Fs on distinct batteries of genes are entirely unknown. Mutant *rbr1* is female gametophyte lethal and shows overproliferation of endosperm tissue [36]. Compromising RBR1 function by overexpression of RBR1-binding viral proteins [37], or RBR1 RNAi silencing constructs [38] leads to overproliferation of leaf epidermal cells. Recently, it was shown that inducible RBR1-silencing also promotes the production of stomata meristemoids [39]. The E2F-RBR1 pathway controls genes in the G1 to S phase transition, and also genes involved in mitosis, such as the plant-specific B-type CDKB1;1. This CDK is specifically expressed in stomata and is required for correct stomatal development in Arabidopsis [40]. Whether the E2F-RB pathway is connected to MAPK signaling in plants is currently unclear.

Signaling via MAPKs share protein components between different pathways, but each specific signal leads to corresponding responses, e.g. MKK4/MKK5 and MPK3/MPK6 are acting in stress and during stomata development [2]. However, currently it is not clear, how the specificity of MAPKs signaling is achieved.

Here, we suggest that protein phosphatases could be part of the regulatory mechanisms to enforce specificity in signal transduction. We show that the KIM-containing PP2Cs AP2C2, AP2C4 and AP2C3 are all MAPK phosphatases with specific expression patterns, where only AP2C3 is expressed in stomata lineage cells from late meristemoids onwards. AP2C3, but not the other PP2Cs tested trigger epidermal cell entering into proliferation and stomata development, consistent with a potential role of AP2C3 in suppressing MAPKs activities in epidermal cells. A specific domain in the phosphatase mediates MAPK interaction and is required for subcellular localization, which is important in triggering ectopic epidermal cell proliferation and differentiation into stomata. Concomitant to AP2C3's effect on epidermal cell fate, we find changes in protein amounts and forms of key cell cycle regulators as well as altered CDK activities. Our results thus integrate plant MAPK signaling and cell proliferation for determination of cellular differentiation.

## Results

### KIM-Containing Protein Phosphatases are MAPK-Phosphatases

KIM-containing PP2Cs, such as MP2C and AP2C1 have been shown to interact with specific sets of MAPK and function as MAPK phosphatases in alfalfa and Arabidopsis, respectively [25], [26]. Thus, we tested the entire Arabidopsis cluster B KIM-containing PP2Cs; AP2C2, AP2C4 and AP2C3 for their ability to interact and co-localize with MAPKs. Interactions in yeast (Figure S2A) proved that indeed all PP2Cs tested were able to interact with MAPKs. These interactions demonstrated selectivity in different combinations of four PP2Cs (AP2C1, AP2C2, AP2C4 and AP2C3) with four MAPKs (MPK1, MPK3, MPK4, and MPK6). MPK4 and MPK6 were preferred interacting partners of AP2C1, AP2C2 and AP2C3, whereas MPK3 and MPK6 showed interaction with AP2C4. MPK1 did not interact with the PP2Cs tested (Figure S2A). Interaction studies in yeast of AP2C3 with 18 Arabidopsis MAPKs revealed that only MPK6 and MKP4 are interacting proteins (Figure S2B). Moreover, MPK6 and MPK4 were identified in a yeast two hybrid screen of an Arabidopsis cDNA library using AP2C3 as bait.

**Figure 1:**
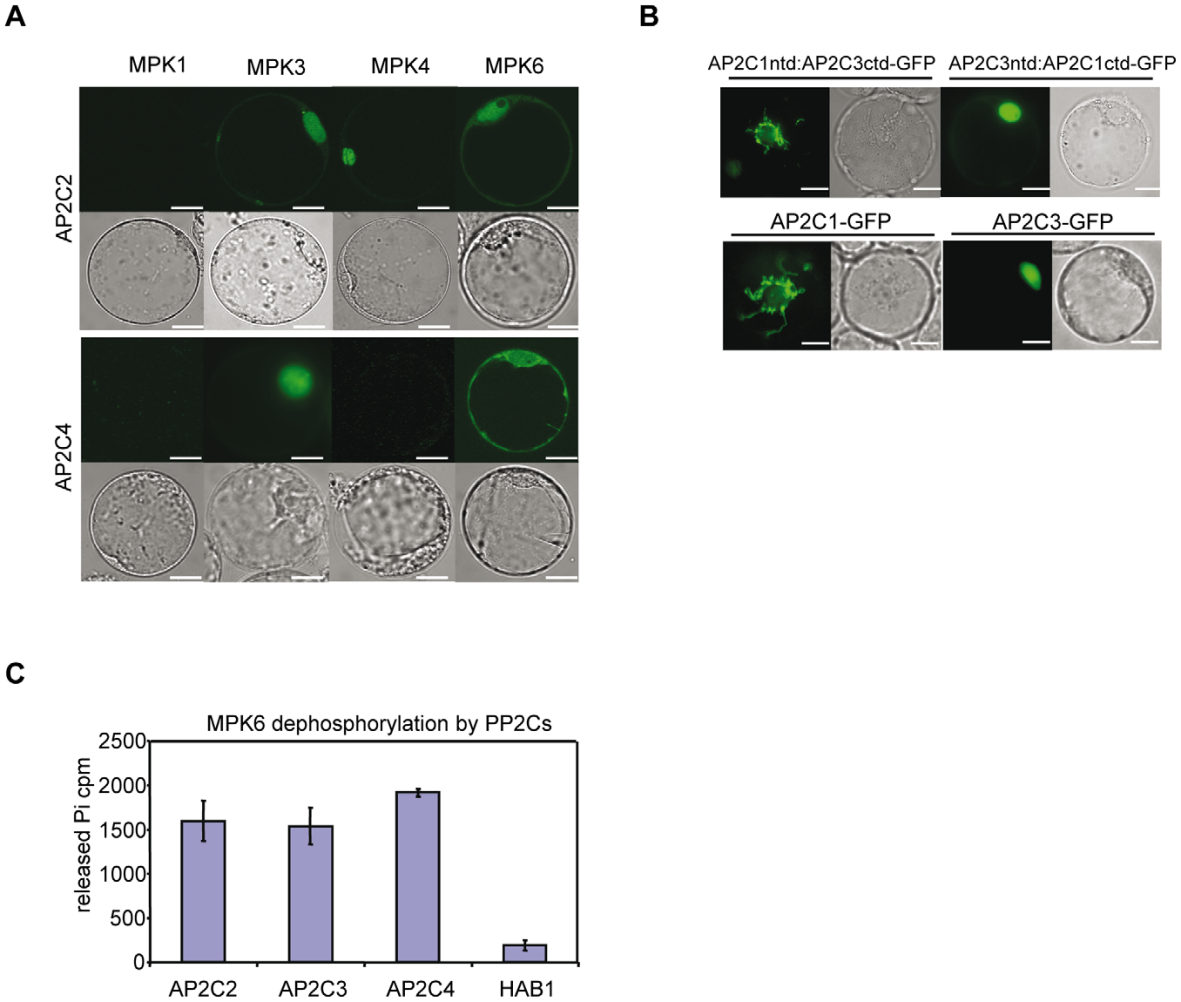
Interaction, inactivation and localization of AP2C3, AP2C2 and AP2C4 with MAPKs. (A) Interaction of AP2C2 and AP2C4 with MPK1, MPK3, MPK4 and MPK6 in protoplasts using bimolecular fluorescence complementation. YFPntd-PP2Cs were co-transfected with YFPctd-MAPKs in Arabidopsis suspension culture protoplasts and the reconstituted fluorescence detected. Fluorescence microscopy and differential interference contrast (DIC) images of protoplasts. Bar  = 10 µm. (B) Localization of AP2C3/AP2C1 chimera proteins and PP2C-GFP in Arabidopsis protoplasts, fluorescence microscopy and differential interference contrast (DIC) images. Bar  = 10 µm. *In vitro* dephosphorylation of recombinant GST-MPK6 by recombinant GST-AP2C2, GST-AP2C3, and GST-AP2C4 proteins. GST-HAB1 is included as control. Error bars indicate standard deviations.

Arabidopsis suspension protoplasts were further used to study protein interactions of PP2Cs with MPK1, MPK3, MPK4, and MPK6 (Figure 1 and Figure S1). Bimolecular fluorescent complementation (BiFC) using split-YFP (yellow fluorescent protein) was performed to assess the PP2C-MAPK interaction and its localization within plant cells (Figure 1A and Figure S1A). AP2C3 as well as AP2C2 interacted with MPK3, MPK4 and MPK6 in protoplasts. Interaction of AP2C3/AtPP2C5 with MPK3, MPK4 and MPK6 is in agreement with recently published data [31]. AP2C4 interacted preferentially with MPK3 and MPK6, but not with MPK4. In agreement with the yeast two hybrid assays, none of the PP2Cs tested showed interaction with MPK1 (Figure 1A and Figure S1A). BiFC signal from AP2C2, AP2C4 and AP2C3 interaction with MPK3 and from AP2C2 and AP2C3 interaction with MPK4 are mostly confined to an area that appears to be the nucleus (based on DIC image), while the BiFC signal from the interaction of these AP2C phosphatases and MPK6 are more diffused.

Colocalization studies of green fluorescent protein (GFP) tagged AP2C3 with red fluorescent protein (mRFP1) tagged MPK3, MPK4 or MPK6 indicated localizations that appeared to be nuclear, both for the tested AP2C3 and the MAPK constructs in co-transfected protoplasts (Figure S1B). These findings are in agreement with recent observations where CFP-tagged AP2C3/AtPP2C5 showed co-localization with YFP-tagged MAPKs [31]. Fluorescence signal from the AP2C4-GFP fusion was very similar to that of AP2C3 and appeared to localize to the nucleus (Figure S1C). AP2C1 and AP2C2 interaction with MAPKs studied by BiFC was observed in the nucleus, AP2C1-GFP and AP2C2-GFP proteins were observed predominantly in cytoplasmic structures that are similar to stromules ([26], and Figure 1B and S1C). Thus, interactions in yeast and *Arabidopsis* protoplasts demonstrated that all PP2Cs tested are indeed MAPK-interacting proteins.

To understand if PP2C and MAPK interaction may result in dephosphorylation of the kinase, AP2C2, AP2C4 and AP2C3 were tested *in vitro* for their ability to dephosphorylate phosphorylated MPK6 protein. We found that AP2C2, AP2C4 and AP2C3 bacterial recombinant proteins efficiently dephosphorylated recombinant phospho-MPK6 substrate (Figure 1C) supporting their role as MAPK phosphatases. In this assay the cluster A PP2C HAB1 [41], which contains no KIM and is not able to inactivate MPK6 [26] was used as a control. These results demonstrate the ability of AP2C2, AP2C4 and AP2C3 to inactivate the MAPK MPK6 and the selectivity of MPK6 towards the KIM-containing PP2Cs AP2C2, AP2C4 and AP2C3.

**Figure 2:**
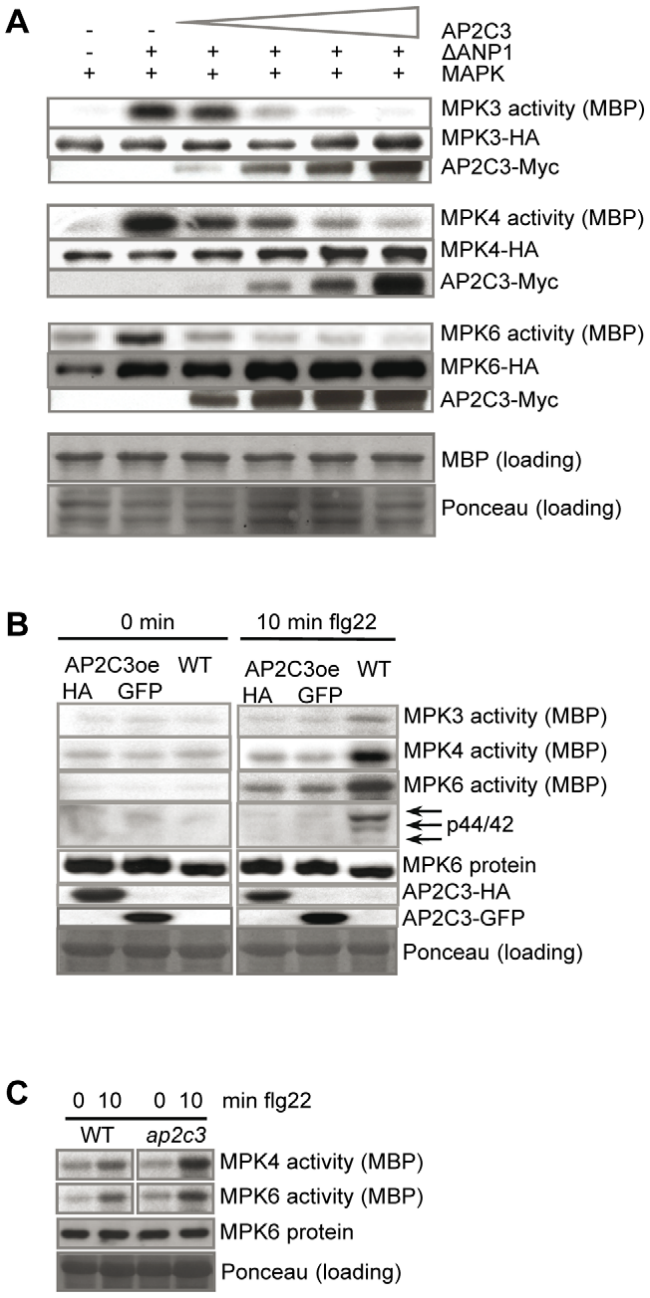
Regulation of MAPKs by AP2C3. (A) Inactivation of MAPKs in cotransfection assays. Protoplasts from an Arabidopsis cell suspension culture were transfected with plasmid DNA coding for MPK3-HA, MPK4-HA, MPK6-HA, ΔANP1-HA and increasing amounts (0, 0.05, 0.1, 0.5, and 1 µg) of AP2C3-Myc plasmid DNA. ΔANP was used as upstream activating MAPKK kinase. MAP kinases were immunoprecipitated and kinase activities were assayed on myelin basic protein (MBP). MBP loading control is demonstrated for MPK6. Proteins were detected by immunoblots using HA and c-Myc antibodies. Loading control of protein extracts is demonstrated by Ponceau-S stained membrane of MPK6 western blot and represents a general loading control. (B) Activation of MAPKs in AP2C3oe (5 dpg) and (C) *ap2c3* (7 dpg) seedlings. Seedlings were treated with 100 nM flg22 for 10 min. and total protein extracts were prepared from cotyledons and hypocotyls. MAPKs were immunoprecipitated with antibodies specific for MPK3, MPK4 or MPK6 and kinase activities were assayed on MBP. Western blot was performed with MPK6 specific antibodies and with p44/42 antibodies against dual phosphorylated MAPKs. AP2C3 proteins were detected by immunoblots using HA or GFP antibodies. Ponceau-S stained membrane represents general loading control and corresponds to MPK6 western blot.

To investigate if AP2C3 functions as a MAPK phosphatase also *in vivo*, transient protein coexpressions and subsequent kinase assays were carried out using Arabidopsis protoplasts. An activated MAPK pathway was reconstituted by cotransfection of constitutively active MAPKKK ΔANP1 plasmid [42] with plasmids containing HA epitope-tagged MPK3, MPK4 or MPK6 (Figure 2A). A progressively more complete inactivation of MAPKs was observed with increasing amounts of AP2C3 protein. These experiments revealed that AP2C3 can efficiently inactivate MPK3, MPK4 and MPK6 (Figure 2A). Inactivation of MAPK is progressively more effective by increasing amounts of the AP2C3 protein.

To investigate if these MAPKs are inactivated also *in planta*, seedlings from AP2C3 overexpression lines (AP2C3oe) and *ap2c3* T-DNA insertion line (see below description of these lines) were studied for MAPKs activities. In plants MPK3, MPK4 and MPK6 can be activated by elicitation with the flagellin peptide (flg22) [42]. The activities of MPK3, MPK4 and MPK6 were strongly suppressed in AP2C3oe lines compared to WT plants demonstrating that AP2C3 was able to block flg22 activation of MPKs (Figure 2B). Western blot with a phospho-specific MAPK antibody detected the active phosphorylated MAPK forms (corresponding to MPK3, MPK4 and MPK6) in flg22 elicitor-stressed WT but not in AP2C3oe seedlings, consistent with the proposed function of AP2C3 as a MAPK phosphatase (Figure 2B). In *ap2c3* seedlings flg22-induced activities of MPK4 and MPK6 were higher comparing to WT (Figure 2C), suggesting that AP2C3 is responsible for regulating the MAPK activation threshold in these plants. This data is in agreement with recently reported observation [31].

Taken together, KIM containing PP2Cs AP2C3, AP2C2 and AP2C4 are MAPK phosphatases, as they colocalize, interact with and dephosphorylate MAPKs *in vitro*, in cells and in plants where AP2C3 can dephosphorylate and inactivate MPK3, MPK4 and MPK6 and is responsible to control MAPK activation in plants.

### AP2C3 Promoter Activity is Confined to Stomata Lineage and Highly Dividing Cells During Early Developmental Stages

The YODA-MKK4/MKK5-MPK3/MPK6 cascade is central to ensure correct and timely stomata initiation. However, how the activity of this cascade can be channeled to induce specific effects on stomata developmental response is puzzling. Negative regulators of MAPKs could be potential modulators of this pathway. As we found that KIM-containing Arabidopsis PP2Cs AP2C1, AP2C2, AP2C4 and AP2C3 are MAPK phosphatases ([26] and Figures 1A, C, 2, S1, S2) we investigated if any of these enzymes may function as regulators in stomatal fate decisions. Assuming that such regulators might be expressed in stomata lineage cells we analyzed promoter::GUS reporter expressions in plants.

**Figure 3:**
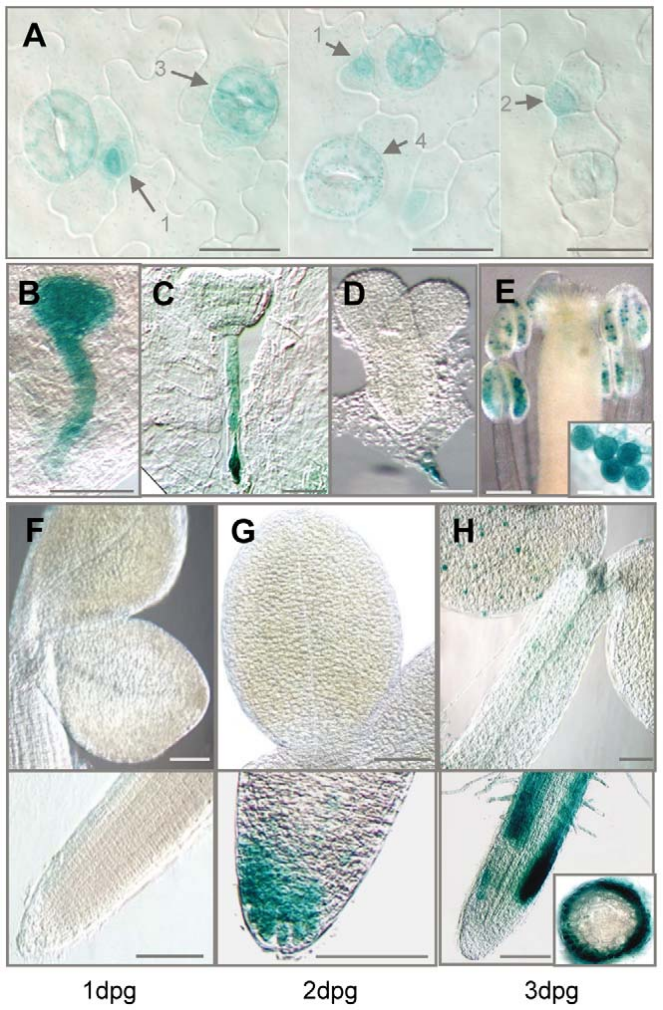
Analysis of AP2C3::GUS expression in plants. Histochemical staining of (A) AP2C3::GUS in late meristemoids (1), GMC (2), young (3) and mature (4) stomata in cotyledons of 5dpg seedlings; (B–D) embryo: (B) globule, (C) heart, (D) torpedo; and (E) anthers (close-up: stained microspores). (F–H) seedlings from 1 till 3 dpg: shoot (upper panel), and root tip (lower panel); inset in (H) in lower panel shows radial section of root. Scale bar (A) = 20 µm, (B–D) = 30 µm; (E) = 250 µm, and close-up image  = 20 µm, (F–H) = 100 µm.

Analysis of promoter activity of all four AP2C protein phosphatases in plants revealed that the promoter of AP2C3, but not of AP2C1, AP2C2 or AP2C4, is active in stomata lineage and guard cells in a number of organs, including cotyledons, hypocotyls and also in true leaves (Figure 3; Figure S3; [26]). AP2C3::GUS signal was detectable in small cells that are part of the stomata lineage, such as late meristemoids, GMC, young stomata and developed stomata (Figure 3A), whereas weaker signal is also detectable in stomata lineage ground cells (SLGC), but not in pavement cells. Interestingly, AP2C3::GUS activity is also present at specific locations during plant development, other than stomata lineage, such as in the globular embryo (Figure 3B) and later it is restricted to the suspensor (Figure 3C–D). GUS expression is found at early stages of root cap development and in patches of root cells that appear to elongate (Figure 3F–H). AP2C3 promoter is highly active in mature pollen (Figure 3E). We could additionally induce expression of *AP2C3* by treatment with cyclohexamide, flagellin (Figure S4D), but not by wounding (data not shown), and this correlates with available *in silico* data (https://www.genevestigator.com/gv/index.jsp) reporting *AP2C3* induction during development and after application of pathogen elicitors.

### Cell Proliferation and Differentiation into Stomata are Induced by AP2C3 Overexpression

To further investigate whether AP2C3 can regulate stomata density, we have isolated and characterized a predictably null mutant containing T-DNA insertion within the 2nd exon of *AP2C3* gene. However, counting the stomata ratios in abaxial epidermis of cotyledons at 3 dpg did not show any difference to WT (Figure S4E, S5). There was also no difference between WT and *ap2c3* in stomata ratios in abaxial epidermis of true leaves. To uncover possible redundancies with other AP2Cs that we have shown above to interact with MAPKs we crossed *ap2c3* with T-DNA insertion lines *ap2c1* [26], *ap2c2* and *ap2c4* mutants to generate double mutants (Figure S4). All double mutants were developmentally normal under conditions tested, and have not shown any significant reduction in stomata ratio in abaxial epidermis of cotyledons (Figure S5). We next made crosses between *ap2c1/ap2c2* with *ap2c4/ap2c3* double mutants to obtain other combinations of multiple mutants. We could identify the triple mutants *ap2c1/ap2c2/ap2c3, ap2c3/ap2c2/ap2c4*, and *ap2c1/ap2c4/ap2c3* among these plants, but also this mutant combination did not reveal changes in stomata numbers (Figure S5 and data not shown).

**Figure 4:**
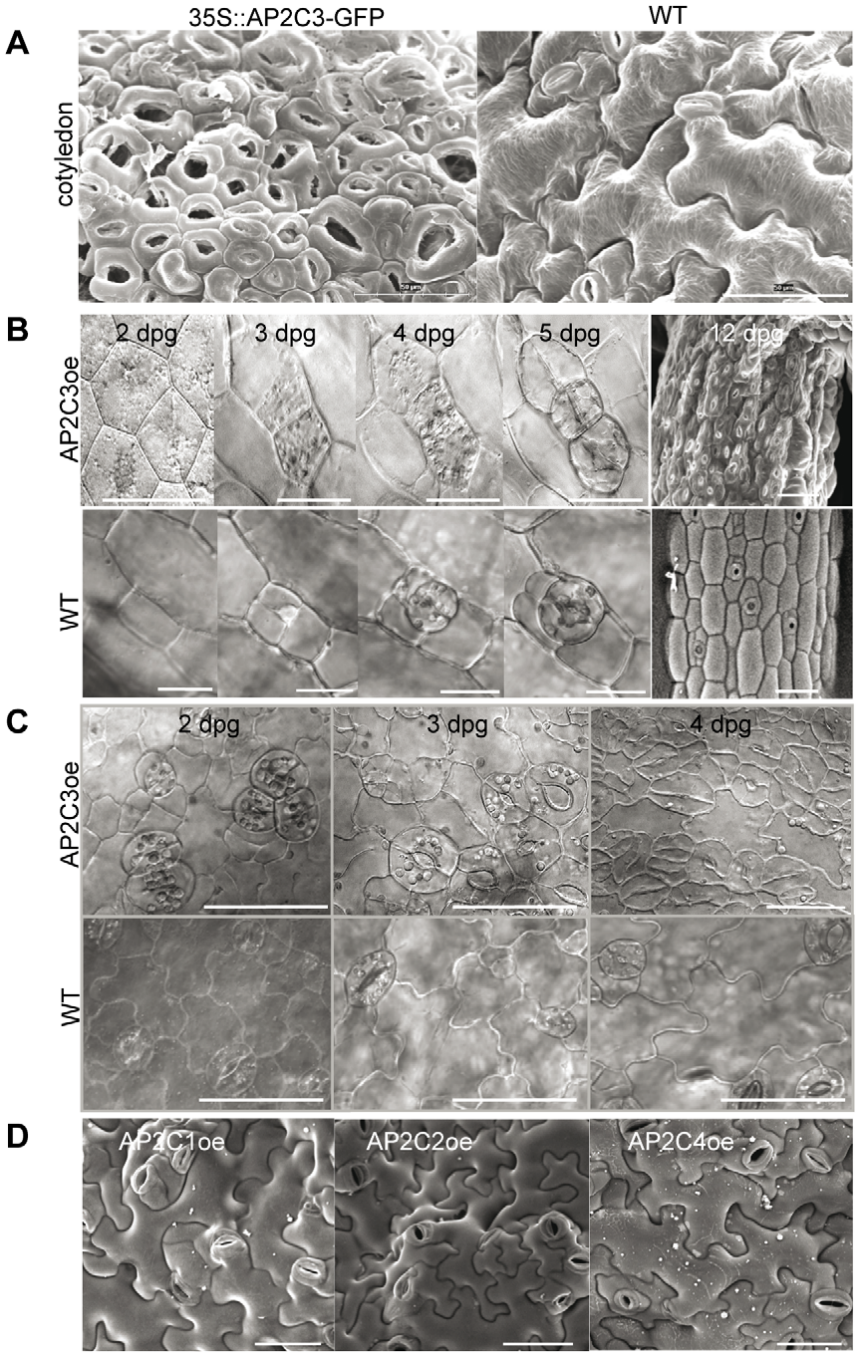
AP2C3 ectopic expression induces stomata clustering and proliferation. (A) Comparison of epidermal surface of cotyledons of WT plants and 35S::AP2C3oe 5 dpg by scanning electron microscopy (SEM). (B) Timing of stomatal cluster formation on hypocotyl of 35S::AP2C3oe plants and WT. DIC images produced from the same place on 35S::AP2C3oe or WT hypocotyl starting from 2 dpg till 5 dpg, Bar  = 20 µm. Hypocotyl at 12 dpg of 35S::AP2C3oe and WT plant (SEM images, Bar  = 50 µm). (C) Stomatal cluster formation on cotyledon of 35S::AP2C3oe seedling compared to WT at 2dpg, 3dpg, 4dpg by DIC images. Bar  = 50 µm. (D) Representative SEM images of abaxial epidermis of 35S::AP2C1-GFP (AP2C1oe), 35S::AP2C2-GFP (AP2C2oe), 35S::AP2C4-GFP (AP2C4oe) at 8 dpg. Bar  = 50 µm.

**Figure 5:**
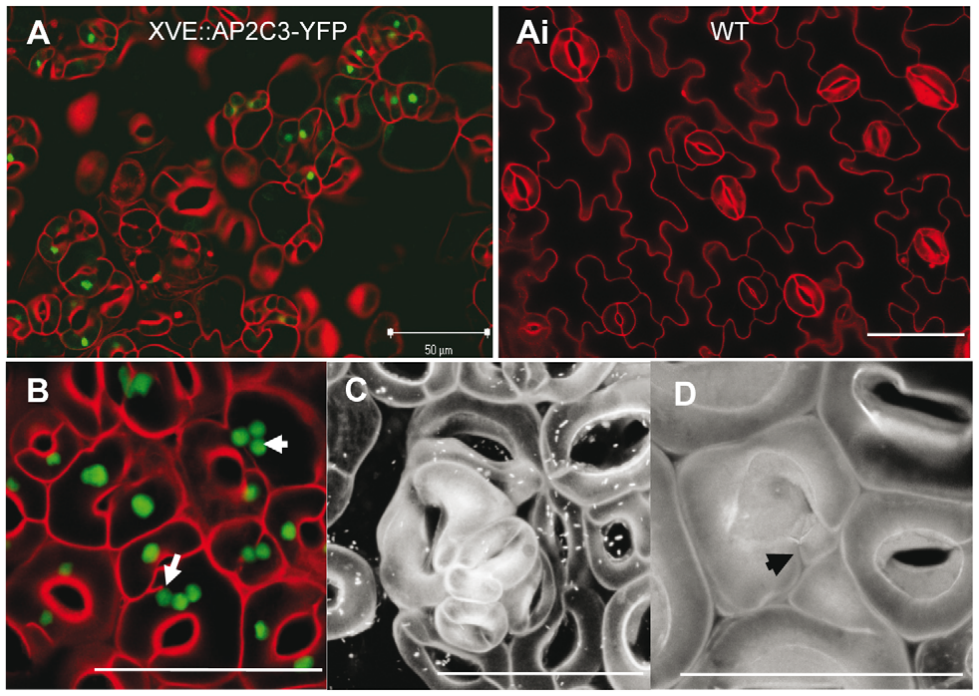
Stomata phenotypes induced by AP2C3 ectopic expression. (A) Stomata cluster formation in true leaves of XVE::AP2C3-YFP seedlings 7 days after induction of AP2C3-YFP expression by 5 µM estradiol. Estradiol was added into ½ MS at 3 dpg. Epidermal surfaces of estradiol-treated line #8 (A) and estradiol-treated WT (Ai) is shown. (B) Multiple nuclei in guard cells in cotyledons of 35S::AP2C3-GFP overexpressing seedling 5 dpg. (C) Stomata clusters and stomata “tumours” in true leaf of 35::AP2C3-HA overexpressing seedling 9 dpg. (D) Emerging of small stomata cell in true leaf of 35S::AP2C3-HA overexpressing seedling 9 dpg. Confocal images, cell walls are stained with propidium iodide (PI). All bars  = 50 µm.

Assuming that inhibitors of MAPKs may act in stomata lineage to prevent epidermal cell differentiation and reinforce the stomata lineage we next tested the ectopic constitutive expression of each of these PP2Cs in Arabidopsis plants and searched for stomata developmental phenotypes. AP2C1 was described previously as MPK4/MPK6 phosphatase and it did not induce stomata related phenotypes by overexpression [26]. Similarly, no changes in stomata development were observed in AP2C2 or AP2C4 overexpressing plants (at least 3 independent transgenic lines were tested for each PP2C) (Figure 4D). We have tested the protein expression of the constructs in plants by Western blotting using antibodies against the HA or GFP tags, and all showed expression of the AP2C constructs, and the expression of AP2C2 and AP2C4 had no effect on stomata development (Figure S6, Figure 4D). A number of different AP2C3 overexpression (AP2C3oe) lines where AP2C3 cDNA or genomic ORFs were placed behind the 35S CaMV promoter and tagged with HA or GFP at C-terminal part have been produced and studied. We also made inducible AP2C3 expression constructs using the estrogen receptor-regulated expression system (XVE-AP2C3). We have tested the expression of the tagged proteins and selected several independent lines for each of these constructs (Figure S7). At least 100 seedlings for each of these constructs and independent lines have been scored for stomata index. All the constitutive overexpression lines of AP2C3 showed a strong overproliferation of stomata, that in extreme cases led to an almost complete conversion of all epidermal cells into guard cells in the cotyledons and hypocotyls (Figure 4A). Clusters of stomata were also observed in true leaves of young seedlings both in constitutive (data not shown) and in estradiol inducible lines after induction with estradiol (Figure 5A). Plants overexpressing AP2C3 also exhibit a dwarf phenotype (Figure S8).

In wild type plants meristemoid cells that originate from epidermal cells by asymmetric cell divisions produce guard mother cells, which divide into two guard cells to create stomata (Figure 4B and [43]). To understand better how stomata phenotypes develop in AP2C3oe plants we monitored stomata cell differentiation from 2 until 12 days post germination (dpg) in cotyledons and hypocotyls of transgenic lines (Figure 4B, C). In cotyledons already by 2 dpg we observed many more and clustered stomata compared to the wild type (WT) Col-0 plants and stomata numbers gradually increased by 4 dpg (Figure 4C) and in extreme cases by 5dpg almost all epidermal cells were converted to stomata in cotyledons (Figure 4A). To study the origin of ectopic stomata cells we chose hypocotyls and tracked the fate of two protodermal cells in time from 2 dpg till 5 dpg (Figure 4B). We observed a perpendicular division to the hypocotyls growth axis of one cell (the lower cell) at 3 dpg, which appeared to be asymmetric as expected for cells entering the stomatal lineage. At 4 dpg the upper cell changed in morphology, and at 5 dpg all three cells divided simultaneously in the same direction, that was perpendicular to the first division to form the 3 pairs of stomata. Apparently, the upper cell went through the fate conversion without stomata lineage divisions. Thus both the one-cell spacing mechanism governing stomata positioning and the requirement for stomata lineage divisions were perturbed in AP2C3oe plants (Figure 4B).

To demonstrate that AP2C3 expression leads to developmental perturbation of epidermal cells, we have studied the lines with inducible AP2C3 expression under the control of estrogen receptor-regulated expression system (XVE-AP2C3). We treated seedlings 3 dpg of six independent lines of XVE-AP2C3-YFP with 5 µM β-estradiol to induce AP2C3 protein expression and observed the stomata phenotypes on the abaxial epidermis of cotyledons at 10 dpg. Clusters of stomata were progressively accumulating up to 10 days in these plants (Figure 5A). Similar experiments were performed with independent XVE-AP2C3-Myc overexpressing lines and gave similar results (data not shown). As demonstrated in Figure 1B, 5B, and Figure S1B AP2C3-GFP is localized to the nucleus. Interestingly, we frequently found cells with 2–3 round shaped structures with GFP signal positioned close to each other within the same cell, suggesting that these cells have multiple nuclei (Figure 5B). We also observed formations of stomata “tumors” or epidermal stomata cell colonies in cotyledons of AP2C3oe plants where the monolayer cell sheet structure that is typical to epidermis of WT plants was lost (Figure 5C, D). Interestingly, some of the guard cells appear to divide to produce small daughter cells, suggesting that even after stomata differentiation, cell proliferation may continue. It is not excluded that new stomata cells are emerging also from already existing stomata (Figure 5D).

### Stomata Lineage Markers are Upregulated in AP2C3oe Plants

**Figure 6:**
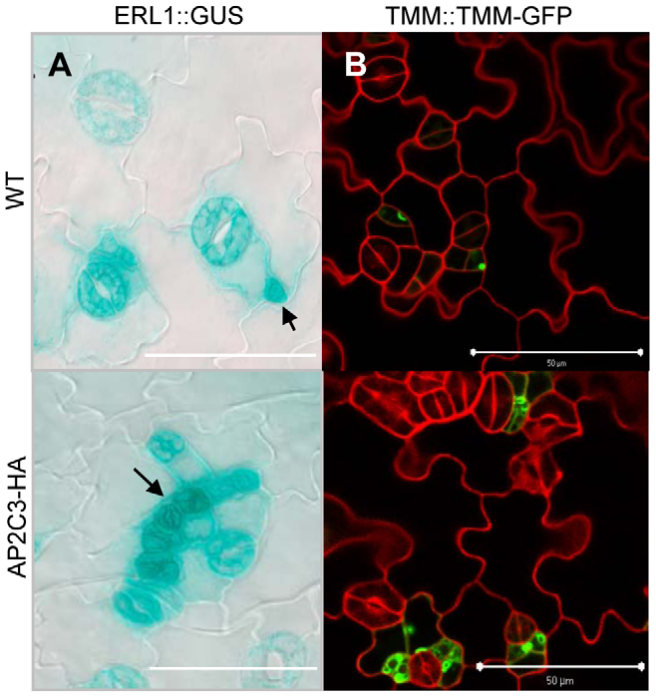
AP2C3-overexpression induces stomata markers ERL1 and TMM. (A) Upregulation of stomata marker ERL1::GUS in AP2C3oe cotyledons. Promoter activity of receptor-like kinase ERL1 is strongly upregulated in 35S::AP2C3-HA in comparison to WT seedlings. Arrows indicate GUS in early cells of stomata lineage at 5 dpg after staining for 4 h. Bars  = 50 µm. (B) Expression of stomata development marker TMM::TMM-GFP in meristemoids of WT and AP2C3oe cotyledons 3 dpg. In AP2C3oe cotyledons meristemoid density is increased and meristemoids are originating at incorrect position (adjacent to stomata). Green: localization of TMM-GFP protein. Confocal images taken under the same settings, cell walls stained with propidium iodide (PI). Bars  = 50 µm.

To investigate the stomata development in AP2C3oe lines we studied the expression of stomata lineage marker proteins and gene activities. The promoter of the receptor-like kinase ERL1 is highly active in meristematic cells and stomata lineage cells but has gradually decreased expression in newly formed stomata lineage ground cells and mature stomata in Arabidopsis [11]. In AP2C3oe seedlings we observed highly upregulated ERL1::GUS and it was localized in dividing cells adjacent to stomata (Figure 6A, S9).

In WT plants the receptor-like protein TMM tagged with GFP is present in cells that enter stomata developmental pathway (Figure 6B and [43]). TMM::TMM-GFP was present in clusters of cells and also strongly upregulated in phosphatase oe plants, based on confocal images (Figure 6B), suggesting that AP2C3oe causes an increase in stomata lineage initiation. TMM-GFP presence in clusters of cells adjacent to developed stomata indicates their competence to enter the stomata lineage at abnormal position and predicts eventual development into stomata clusters (Figure 6B).

**Figure 7:**
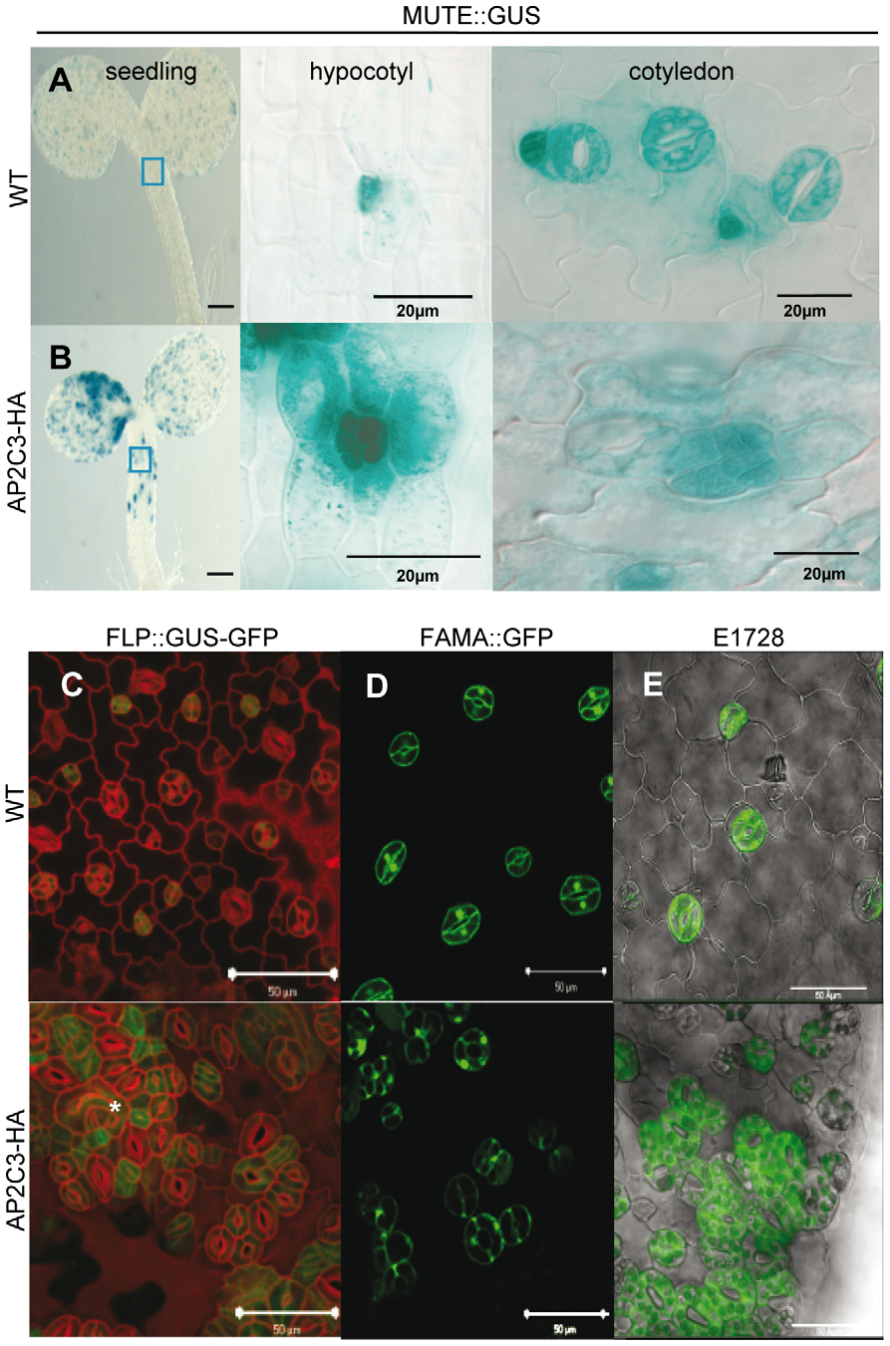
AP2C3-overexpression induces stomata markers: MUTE, FLP, FAMA and E1728. (A, B) Expression of meristemoid/GMC-specific MUTE in WT (A) and in AP2C3-HA overexpressing (oe) (B) line 5 dpg: MUTE expression in seedlings, hypocotyl and cotyledon. (C, D, E) The stomata lineage cells of WT and AP2C3oe lines express stomata pathway markers: FLP::GUS-GFP (C) in true leaves of WT and AP2C3oe plants 9 dpg. Asterisk indicates epidermal cell that acquired guard mother cell (GMC) identity. Green: GFP fluorescence. Confocal images, cell walls stained with PI. Bars  = 50 µm. GMC/guard cell marker FAMA (D), and guard cell marker E1728 (E). Promoter activities of FAMA::GFP and E1728::GFP show enhanced expression in cotyledons 5 dpg in AP2C3-HA overexpressing (oe) seedlings. Green: GFP fluorescence. Bars  = 50 µm.

To investigate, whether the ectopic stomata induced by AP2C3oe goes through the established developmental transitions, we have studied markers representing each of these developmental stages. MUTE was shown to regulate GMC/stomata differentiation and MUTE::GUS expression is observed in late meristemoids/GMCs/immature stomata cells (Figure 7A and [16]). In AP2C3oe seedlings MUTE::GUS staining was much stronger compared to WT (Figure 7A, B). In seedlings this signal was most prominent in the cotyledons and the upper part of the hypocotyl (Figure 7A, B), where during seedling development cell proliferation is maintained for a longer time [44]. In the lower part of the hypocotyl, where predominantly endoreduplication is occurring in WT plants [44], [45]; MUTE::GUS staining was not observed in AP2C3oe seedlings; as well as no stomata clusters were detected in this part of the seedling (Figure 7A, B). This observation supports the assumption that cell proliferation and stomata developmental pathways are related.

The R2R3-type MYB transcription factor FLP is expressed in late guard mother cells (GMCs) and in young developing stomata in wild type plants [46]. In AP2C3oe lines clusters of FLP::GUS-GFP positive cells were surrounding the fully developed stomata, suggesting that these cells can gain stomata identity by passing through the guard mother cell stage (Figure 7C).

Promoter activity of GMC/stomata marker FAMA (Figure 7D) and the guard cell marker E1728 [15], [16] (Figure 7E) were observed in guard cells of AP2C3oe lines confirming their stomata cell identity.

Thus the data presented demonstrates that in AP2C3oe seedlings stomata lineage marker expression is upregulated and more stomata lineage cells than in WT are observed in AP2C3oe lines. Stomata lineage cells are originating adjacent to stomata thus breaking the patterning. Our data suggests that in phosphatase overexpressing lines cells acquire the stomatal lineage by inducing the early components of the signaling cascade as well as specific transcription factors.

### Modulation of Key Cell Cycle Proteins and CDK Activities in AP2C3oe Plants

**Figure 8:**
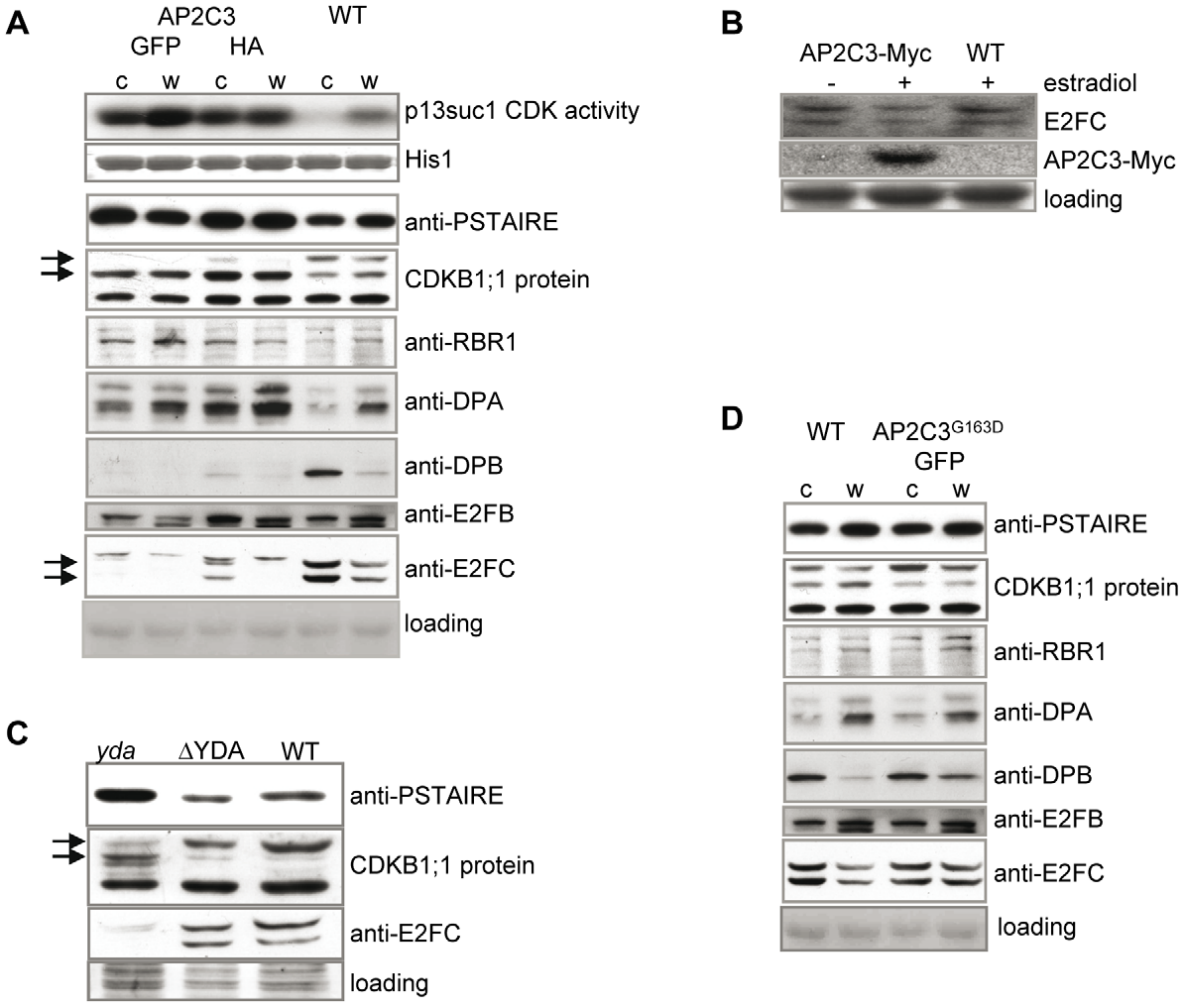
AP2C3 overexpression induces cell proliferation. (A) Analysis of CDK activity, CDK proteins and protein levels of CDKB1;1, RBR1, DPA, DPB, E2FB, E2FC, and PSTAIR in 35S::AP2C3-GFP (indicated as GFP) and 35S::AP2C3-HA (indicated as HA) lines compared with wild type plants (WT). CDK activity correlates with CDKB1;1 protein mobility shifts in SDS-PAGE of protein extracts isolated from both AP2C3oe lines (AP2C3 tagged with GFP and AP2C3 tagged with HA) compared with WT in seedlings 5 dpg; total protein extracts from: c - cotyledons, w – whole seedlings. Total CDKs were precipitated with p13Suc1 sepharose. Kinase activity was assayed on histone 1 substrate. Western blotting was performed with CDKB1;1, RBR1, DPA, DPB, E2FB, E2FC and PSTAIRE antibodies. Anti-PSTAIRE detects CDKA. (B) Induction of AP2C3 expression by estradiol affects abundance of E2FC protein. E2FC protein levels were detected by specific antibody in estradiol-induced AP2C3-Myc overexpressing seedlings 5 dpg. Seedlings were grown with (+) or without (−) 5 µM estradiol. AP2C3 induction demonstrated by Western blotting with anti-Myc antibody. (C) Western blotting of cell cycle marker proteins PSTAIRE, CDKB1;1 and E2FC in *yda*, ΔYDA and WT (whole seedlings 5 dpg). (D) Abundance of cell cycle marker proteins in AP2C3^G163D^ overexpressing seedlings compared with WT, 5 dpg. AP2C3^G163D^ is a mutant protein with ∼90% reduced phosphatase activity. Total protein extracts from: c - cotyledons, w – whole seedlings.

Our observation that many more epidermal cells are induced to divide in AP2C3oe lines suggested a possible link to cell cycle control. It has been shown that overexpression of E2FA transcription factor together with its dimerisation partner DPA can lead to cell overproliferation specifically around stomata [47]. Therefore, we determined whether cell proliferation is deregulated in AP2C3oe lines. Initially we measured CDK activity through purification of total CDK (CDKA and CDKB) through its interaction with p13^Suc1^ beads [48], using extracts of cotyledons, which have a low proportion of dividing cells in WT plants, and also from whole seedlings with a higher proportion of dividing cells. WT cotyledons show lower CDK activity compared to the whole seedling, but in AP2C3oe lines the CDK activity was much higher in both samples indicating active cell proliferation in the cotyledon (Figure 8A). We also determined the protein levels of CDKA (with PSTAIRE antibody) and CDKB1;1 within these samples. In WT seedlings where the CDK activity was reduced also the CDKA protein level is strongly reduced in the cotyledons compared to the amount detected in the entire seedling. However, in AP2C3oe a high CDKA level is maintained in the cotyledon. It was suggested that CDKA levels correspond to proliferation capacity of plant cells [49], and thus it appears that in AP2C3oe this proliferation competence is maintained. CDKB1;1 showed a characteristic change from a low mobility form indicative of low cell proliferation to a higher mobility form associated with increased cell proliferation (Figure 8A). In WT seedlings, we could detect three distinct mobility forms of CDKB1;1, where the low mobility form was more abundant in the cotyledon sample with low cell proliferation activity. However, this low mobility CDKB1;1 form was almost undetectable in the AP2C3oe lines, indicating that this CDKB1;1 form is associated with exit from cell proliferation, which is inhibited by the overexpression of AP2C3 (Figure 8A).

The RBR1-E2F transcriptional regulatory switch plays important roles in the exit from cell proliferation [35]. Therefore, we decided to check the abundance of proteins within this pathway. E2FB is suggested to play a positive role in cell proliferation, but it was not significantly altered in AP2C3oe lines (Figure 8A). However, E2FC, DPA, DPB and RBR1 showed a marked change in abundance in AP2C3oe plants (Figure 8A), indicating the involvement of this regulatory mechanism in enhanced cell proliferation in AP2C3oe lines. E2FC is a repressor type E2F associated with tissues with low cell proliferation such as dark-repressed meristems [50]. We could detect two major forms of E2FC in the WT, but in AP2C3oe lines both forms and in particular the higher mobility form was strongly diminished (Figure 8A). Reduced E2FC protein level was also observed in plants with inducible AP2C3 overexpression after estradiol application, demonstrating that depletion of E2FC is dependent on AP2C3 protein overexpression (Figure 8B). DPB and DPA are proteins that associate with E2Fs [35] but functional difference among these paralogous proteins has not yet been fully explored. DPB was shown to associate with E2FC in a repressor complex [51], while DPA coexpressed with E2FA and E2FB were shown to stimulate proliferation [52], [53]. Correspondingly, in AP2C3oe plants DPB is downregulated similar to E2FC, while DPA is upregulated (Figure 8A). RBR1 is a repressor of E2Fs, but surprisingly the protein amount is enhanced in AP2C3 cotyledons and whole seedlings compared to WT plants (Figure 8A). This finding may correlate with RBR1 expression in proliferating cells, such as root meristems [54], and may function to maintain proliferation competence.

**Figure 9:**
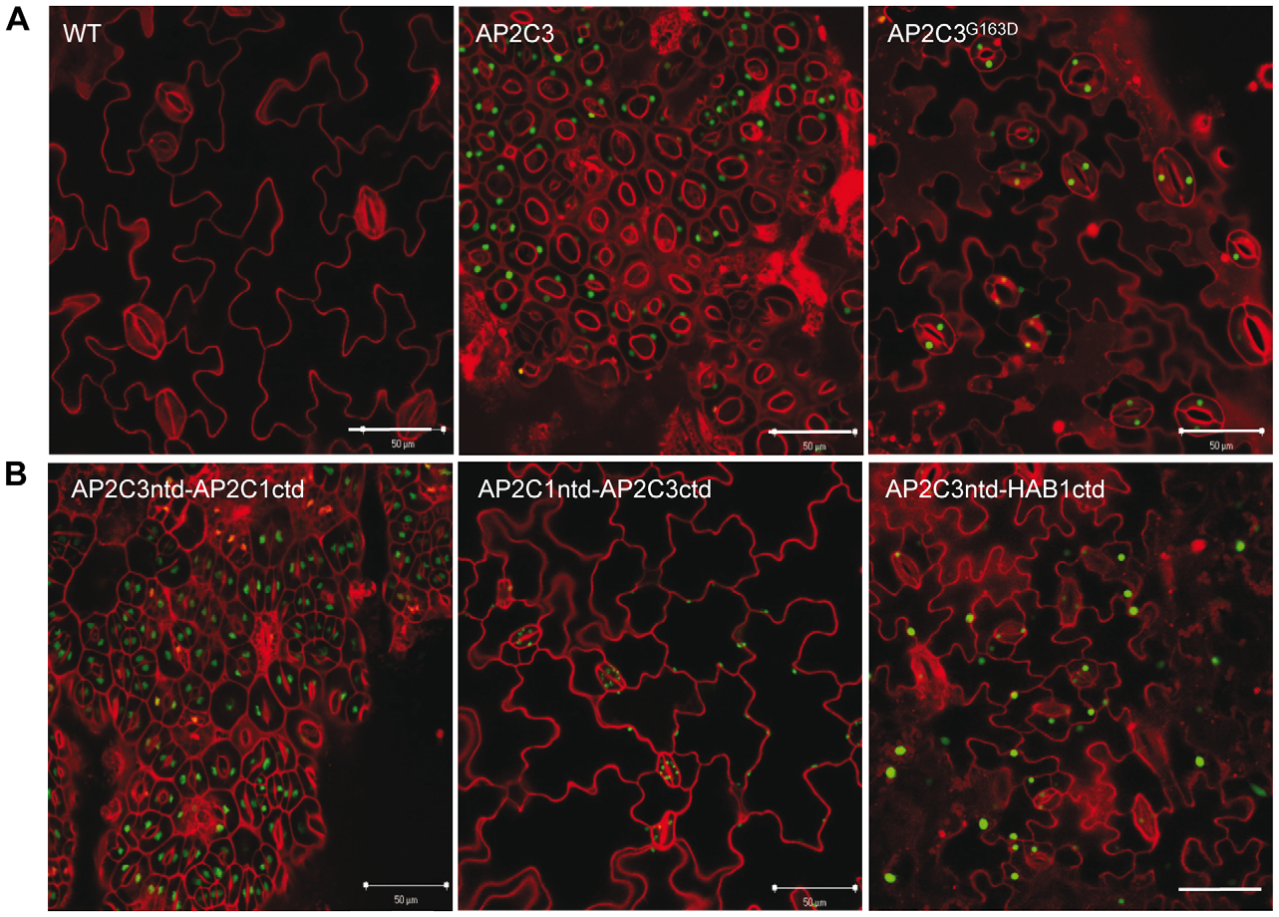
Phosphatase activity of AP2C3 and AP2C3 N-terminal domain (aa 1–135) are essential for stomata cluster formation. (A) Overexpression of AP2C3^G163D^ is not inducing stomata overproduction. C-terminally GFP-tagged AP2C3 or AP2C3^G163D^ proteins were expressed under the control of 35S promoter in Arabidopsis plants. (B) Chimera protein containing N-terminal domain (ntd) of AP2C3 and C-terminal domain (ctd) of AP2C1 is inducing stomata overproduction, while chimera protein ntd of AP2C1 and ctd of AP2C3 is not inducing more stomata when overexpressed in plants; nuclear localized chimera protein between AP2C3ntd and HAB1ctd is not inducing excessive stomata. C-terminally GFP-tagged AP2C3ntd-AP2C1ctd, AP2C1ntd-AP2C3ctd and AP2C3ntd-HAB1ctd proteins were expressed under the control of 35S promoter in Arabidopsis plants. Confocal images, red: propidium iodide staining; green: green fluorescence of fusion proteins with GFP. Bars  = 50 µm.

To investigate if changes in regulation of cell proliferation are related to inactivation of MAPK cascade we studied MAPKKK YODA modified lines. Mutant *yda* exhibits excess of stomata in cotyledons [12], while plants expressing delta N-YDA (ΔYDA) leading to constitutively activated MAPK pathway exhibit formation of pavement cells only with rare asymmetric cell divisions and no differentiation of stomata [4], [12]. We found that *yda* plants have strongly reduced E2FC protein amounts, and enhanced levels of PSTAIR-containing CDKs. In contrast, both cell cycle markers were unchanged in cotyledons of ΔYDA plants, where only rare cell divisions take place (Figure 8C). Overexpression of an inactive version of AP2C3 (see below) did not lead to stomata clusters (Figure 9A) and no changes in cell cycle regulators were detected (Figure 8D). Taken together these results show integration of MAPK signaling and the cell cycle through regulation of CDK activity and the RBR1-E2F pathway.

### The AP2C3 Phosphatase Catalytic Activity and Localization to the Nucleus is Essential in Stomata Cluster Formation

To establish whether the catalytic activity of AP2C3 phosphatase is needed for induction of ectopic cell differentiation into stomata, we overexpressed a catalytically inactive version of AP2C3 carrying the mutation G163D (Figure S10), which is corresponding to the null mutation G139D of AtPP2CA [55]. Phosphatase activities of recombinant glutathione S-transferase (GST) fusion protein AP2C3-G163D was monitored by measuring the release of free phosphate from [^32^P] phospho-casein substrate *in vitro* and confirmed that this mutation is abolishing catalytic activity of AP2C3 (Figure S10).

Overexpression of AP2C3 G163D-GFP in plants was localized similarly as AP2C3-GFP protein to the nucleus as shown by GFP fluorescence, however it neither induce stomata clusters (Figure 9A) nor alterations of cell cycle markers (Figure 8D). This demonstrates that the enzymatic activity of AP2C3 phosphatase is essential for induction of excessive stomata in plants and modulation of cell cycle proteins. It also indicates that the stomatal phenotype in AP2C3oe is not likely to be due to a dominant-negative effect caused by the overexpression of the AP2C3 protein.

The inactivation of MPK3, MPK4 and MPK6 by AP2C3 is confined to the nucleus, where both proteins colocalize and interact as shown by protein fluorescence from fluorescent tags and by bimolecular fluorescence complementation (BiFC) assays (Figure 1B; S1A, B). Nuclear localization of AP2C3 in seedlings of transgenic plants (Figure 5A, 5B, 9A) provides further support that the signaling cascade is controlled by AP2C3 in the nucleus. AP2C3-GFP localization is different to the AP2C1-GFP protein, which is predominantly localized to the cytoplasmic structures (stromule) (Figure 1B), even though AP2C1 interaction with MPK4 and MPK6 is detected in the nucleus by the BiFC [26]. AP2C1 and AP2C2, but not AP2C3 or AP2C4 proteins contain a putative plastid targeting sequence in their N-terminal region (http://www.helmholtz-muenchen.de/en/mips/). Both AP2C3 and AP2C1 share sequence homology in the catalytic domain and dephosphorylate MPK6 (Figure 1C and [26]), however, AP2C1 overexpression did not induce stomata phenotype in plants (Figure 4D and [26]).

To investigate the attribute determining the specificity of AP2C3 function in stomatal differentiation and stomata cluster induction, chimeric constructs consisting of N-terminal domain (ntd) swaps between the AP2C1 and AP2C3 were produced and overexpressed in Arabidopsis plants. Overexpression of the chimera AP2C3ntd:AP2C1 C-terminal domain (ctd)-GFP (containing aa 1–135 of AP2C3 and aa 147–396 of AP2C1) induced excessive stomata differentiation and clustering in epidermis of hypocotyls, cotyledons and emerging true leaves, while overexpression of AP2C1ntd:AP2C3ctd-GFP (containing aa 1–146 of AP2C1 and aa 136–390 of AP2C3) resulted in a WT appearance (Figure 9B), similar to AP2C1 overexpression (Figure 4D). In plants AP2C3ntd:AP2C1ctd-GFP protein is localized to the cell nucleus and AP2C1ntd:AP2C3ctd-GFP is localized to cytoplasmic structures (Figure 9B). Chimera protein localization triggered by the N-terminal exchange was also confirmed in protoplast experiments (Figure 1B). These results demonstrate that the N-terminal domain of AP2C3 determines protein localization to the nucleus and this is required for the protein phosphatase to exert its function in conversion of epidermal cells to stomata and induction of cell divisions. To establish whether AP2C3 shows target-specific dephosphorylation and its requirement for excessive stomatal induction, the chimera between AP2C3 and another nuclear localized PP2C from cluster A in Arabidopsis PP2C family HAB1 [56] was created. HAB1 is a nuclear localized PP2C, which is however unable to interact with or to inactivate MAPKs [26] (Figure 1C and unpublished data). AP2C3ntd:HAB1ctd-GFP (containing aa 1–135 of AP2C3 and aa 197–511 of HAB1) was constitutively expressed in transgenic plants and identified to be localized to the nucleus as demonstrated by GFP fluorescence. Plants overexpressing AP2C3ntd:HAB1ctd-GFP demonstrated normal stomata development in contrast to plants overexpressing AP2C3ntd:AP2C1ctd-GFP (Figure 9B).

Taken together, this data shows that enzymatic activity and nuclear localization of AP2C3 is essential in control of stomatal initiation and that the ctd of AP2C1 and AP2C3 MAPK phosphatases are complementary in the function to induce stomata. Moreover, the nuclear PP2C AP2C3 but not HAB1 is inducing stomata phenotype in plants when overexpressed, suggesting that both nuclear localization and ability to inactivate MAPKs are essential for induction of stomata phenotype in plants.

## Discussion

The initiation of stomata developmental pathway from pluripotent protodermal cells within the epidermis as well as the subsequent steps of stomata differentiation to mature stomata relies on the action of bHLH transcription factors SPCH, MUTE and FAMA [57]. This cell intrinsic transcriptional program is opposed by cell extrinsic developmental or environmental cues to determine both the frequency of stomata initiation, and thus the stomata density and the patterning that always separates stomata by at least one pavement cell [3], [58]. Secretory peptide ligands EPF1 and EPF2 produced by cells within the stomata lineage lead to the stimulation of signaling mediated by putative receptors TMM and/or ERECTA family members and a MAPK module YODA-MKK4/MKK5/-MPK3/MPK6 as well as MKK7/MKK9 [5], [59]. It appears that this signaling activity is opposing the transcriptional program, thus blocking the stomata development at two critical points i.e. at the initiation and during the inhibition of stomata neighbors to become stomata. A positive regulator of stomata density is a mesophyll-secreted peptide STOMAGEN (EPFL9) [10], [60], [61], which appears to oppose EPF1 and EPF2 actions, possibly through ligand competition [61]. Collectively, the negatively acting ligands EPF1/EPF2, and the signaling components ERECTA, TMM and YODA are abundantly expressed in cells within the stomata lineage [7], [8], [9], [11], [12], [43]. Since both the ligand and the signaling components of the pathway are available, what prevents the occurrence of autocrine stimulation of MAPK pathway and thus the inhibition of stomata developmental program in these cells? It is possible that stomata-specific expression of a factor that inhibits the signaling pathway tunes the sensitivity and responsiveness of the stomata lineage cells. Typically, MAPKs are inactivated though dephosphorylation by protein phosphatases. Reasoning that regulation of stomata signaling by MAPK-inactivating enzymes could allow dampening the responsiveness of stomata precursor cells to signals that promote the exit from stomata amplifying cell divisions towards differentiation into pavement cells, we searched for potential candidates among the MAPK-interacting AP2C phosphatases. We identified AP2C3 that is expressed within the stomata lineage cells and found that AP2C3 is potent to stimulate stomata amplifying divisions and to maintain cells in the stomata differentiation pathway. The ability and remarkable specificity of AP2C3 to channel the pleiotropic MPK3 and MPK6 pathway towards the stomata development may rely not only on its stomata lineage specific expression, but also on its definite docking interaction with the MAPKs, as well as its particular localization within the cell.

### AP2C3 Functions in Tuning the Stomata Signaling Pathway to Maintain the Stomata Lineage

The cluster B within the Arabidopsis PP2C-type phosphatases has a predicted **k**inase **i**nteraction **m**otif (KIM) and, therefore, its members are potent to interact with MAPKs [26], [29], [31]. Developmentally regulated gene activities of four AP2Cs members of this subgroup in Arabidopsis revealed distinct patterns in leaves, where only AP2C3 was found to be expressed within the cells of the stomata lineage, from the meristemoid stage onwards. AP2C3 promoter activity suggests strongest expression in young stomata and in subset of meristemoids and stomata, whereas weaker expression is also detectable in stomata lineage ground cells (SLGC), but not in pavement cells. The stomata-lineage-specific AP2C3 gene activity could be observed on the epidermis of a number of organs including cotyledons, hypocotyls and true leaves, indicating that AP2C3 might be a general regulator of stomata development in several organs. Correspondingly, AP2C3 ectopic expression had the same effect of converting epidermal cells into stomata in all these locations. This is consistent with the action of AP2C3 at the MAPK level, whereas the action of signaling components at the receptor level, i.e. ERECTA and TMM is known to be tissue specific and context dependent [11], [43].

The expression of MAPK phosphatases are often connected to the signaling pathways they regulate. In animal cells MAPK phosphatases are rapidly induced by MAPKs without a need for *de novo* protein synthesis for their transcription [62], [63]. Similarly, we have found that a MAPK phosphatase AP2C1 is rapidly and locally induced within minutes after wounding around the wounding site to inactivate wound-induced MAPKs [26]. It is possible that AP2C3 expression is responding to the activity of YODA pathway in the stomata, but this has to be verified. Crossings between AP2C3oe and ΔYDA lines could provide further evidence for the involvement of AP2C3 to inactivate the YODA-induced pathway. AP2C3 interacts with and inactivates not only MPK3 and MPK6, the two MAPKs that are implicated in stomata development, but also MPK4, a MAPK that is expressed in guard cells [64]. AP2C3 expression in mature guard cells suggests that AP2C3 might have a function also in stomata aperture control in these cells. This has been recently demonstrated for a knock out mutant of AP2C3/AtPP2C5 [31]. Though the authors did not explicitly discuss the findings, correlation of an increased MPK4 activation in *ap2c3* mutant lines and MPK4 expression in stomata suggests that MPK4/AP2C3/AP2C1 module regulates stomata aperture. At the same time, AP2C3 may also control stomata aperture in response to pathogens through MPK3 [65], [66].

To address whether AP2C3 inhibition of MAPK activities is sufficient to direct stomata development, we ectopically expressed all four KIM-domain containing AP2C phosphatases under the control of a constitutive and for AP2C3 also inducible promoters. Only AP2C3 led to a dramatic change in cell fate that in extreme cases resulted in the conversion of all epidermal cells into stomata. Even though, several of these MAPK phosphatases can interact with and inactivate MPK3 and MPK6 ([26], [31]; this publication and data not shown), only AP2C3 has the potential to influence the stomata developmental pathway. This suggests that the role of AP2C3 is to inactivate MAP kinases in stomata cell lineage and through this regulation to help maintaining the stomata developmental program. Furthermore, AP2C3 expression in meristemoids could also regulate the rounds of amplifying divisions of stomata stem cells. This is also suggested by the stomata clustering phenotype that is observed in leaves of a putative enhancer trap line of AP2C3::AP2C3-GFP where AP2C3 protein levels were increased within its own expression domain (data not shown).

Unfortunately, it was not possible to study AP2C3 functions in stomata lineage by reverse genetics as T-DNA insertion mutant line did not show a statistically significant change in stomata density. The lack of mutant phenotypes is not unusual for gene families of signaling components as reported also for other members of the MAPK signaling pathway [4]. Thus double and triple mutant combinations of *ap2c3* and other members of the KIM-containing AP2Cs were created, but also these did not show alterations in stomata density. However, *ap2c3* and *ap2c3/ap2c1* mutant plants have been recently reported to demonstrate enhanced ABA-insensitive phenotypes during germination and increased stomata aperture along with enhanced ABA-induced MAPK-activities [31]. Even though these signaling components have distinct expression patterns and specific roles in WT plants, lack of stomata developmental phenotypes in mutant plants suggests overlapping or compensatory functions related to stomata development with other protein phosphatases. In several cases plant mutant phenotypes were found to be masked by the compensatory change in expression domains of genes with redundant functions, e.g. *PINs* [67] or *ACS* members [68]. This limitation can be overcome by constitutive or targeted ectopic expression; the latter has been shown to be informative in dissecting the role of MAPKs during stomata development [5]. Studying AP2C3 action during specific transitional stages and in stomata lineage mutant backgrounds should reveal its function in more detail.

### AP2C3 can be a Specificity Determinant for Pleiotropic MAPK Signaling Pathways

It is intriguing how the signaling specificity of different MAPK cascades sharing the same MAPKK-MAPK module can be achieved. One possibility may be provided through the action of protein phosphatases. We have shown that ectopically expressed AP2C3 has a surprising specificity to divert epidermal cells into the stomata lineage and that the other MAPK-interacting AP2Cs were not able to exert an equivalent function in stomata cell fate control. What determines the specificity of AP2C3 in stomata developmental decisions? It appears that beside specific gene expression domains, protein-protein interactions as well as intracellular co-localizations with the MAPKs are major determinants of signaling specificity.

The presence of a kinase interaction motif (KIM) at the N-terminal non-catalytic part of four Arabidopsis cluster B PP2Cs suggests that these phosphatases should target similar substrates [29]. KIM is a short positively charged amino acid sequence (K/R_(3–4)_, X_(1–6)_, L/I,X,L/I), which was originally identified in upstream regulators of MAPKs, the MAPK kinases in yeast and animals, and later was also found in plant MAPKKs [28], [69]. The same motif is used for the interaction of MAPK phosphatases with their substrates MAPKs and is responsible for docking to their C-terminal negatively charged docking site. Previously, we demonstrated that MAPK phosphatase AP2C1 is interacting with MAPKs via KIM where mutations within KIM of AP2C1 abrogated phosphatase-MAPK interactions [26]. Similarly, the same mutations in AP2C3/AtPP2C5 abolished its interaction with MAPKs [31]. Nevertheless, it was still unclear if other KIM-containing AP2Cs are also MAPK phosphatases. Our data here show that indeed all cluster B KIM-containing PP2Cs are functional MAPK phosphatases. All of them interact with MAPKs, albeit with apparently different affinity, and all are able to dephosphorylate specific MAP kinases. Lack of KIM in PP2Cs, such as HAB1 or ABI2, and their inability to interact and inactivate MAPKs [25], additionally supports our assumption that KIM-containing AP2Cs are true switches of MAPKs. Interestingly, the interaction of all cluster B PP2Cs were found to be directed towards MPK3, MPK4, and MPK6, kinases that mediate stress and also stomata developmental signaling. All four KIM-containing phosphatases are interacting with MPK6, whereas interaction with MPK3 or MPK4 differs. Targeting of MPK6 with multiple phosphatases might relate to the pleiotropic activation by many signals, whereas the activation of MPK3 and MPK4 appears to be more selective [2], [70]. Subtle variations within the KIM domain as well as in adjacent non catalytic N-terminal extension of AP2Cs could be important to modulate the affinity towards different MAPKs. This in turn may determine the duration of the activity of the different MAPKs, which has been shown to impact the signaling output in animal cells, e.g. to choose between proliferation vs differentiation [71]. It is not unusual that the same or overlapping sets of MAPKs are being activated by diverse upstream inputs, yet they generate very different responses. The activation of MPK3 and MPK6 by a multitude of signals is one example, where the specificity is partially achieved by coupling the activation to different upstream signaling components. For example, during stomata development MPK3 and MPK6 are likely activated by YODA and MKK4, MKK5 [4], as well as MKK7, MKK9 may also activate these MAPKs [5], while during pathogen-induced signaling the same MAPKs can be activated by MEKK1 and MKK1, MKK4, MKK5 [72]. Since the MAPK phosphatases may compete with MAPKKs for the same interaction domain on MAPKs, it is an intriguing possibility that AP2C phosphatases could provide another level of MAPK specificity control.

### AP2C3 Specificity towards MAPK Signaling Pathways is Dependent on the Intracellular Localization

Differential induction and localization of MAPK phosphatases raises the intriguing possibility to set distinct activation patterns for cytoplasmic and nuclear pools of MAPKs, and can add to the repertoire of signaling responses that determine cell fate decisions [73]. MAPK phosphatases are in many cases nuclearly localized [74]. We show that nuclear localization of AP2C3 is essential for induction of excessive stomata development. Contrary to AP2C3, the N-terminal parts of AP2C1 and AP2C2 contain a putative plastid targeting sequence. Even though both AP2C1 and AP2C2 can interact with MAPKs in the nucleus, as shown by the BiFC assay in plant cells, the major pool of AP2C1 and AP2C2 is localized to plastid-related structures (this work; [26], [31]; Meskiene, unpublished). Domain-swapping of N-terminal non-catalytic domains between AP2C3 and AP2C1 suggests that C-terminal catalytic domains of these phosphatases can act redundantly, while the N-terminal part has evolved specific properties, such as the interactions with MAPKs and the localization either in the nucleus and/or in the plastids.

### AP2C3-Overexpression promotes Cell Proliferation and Entry into Stomata Pathway

Initiation and proliferation of meristemoids are suggested to be negatively regulated by the MAPK cascade YDA → MKK4/MKK5/MKK7/MKK9 → MPK3/MPK6 [5], [32]. High activation of MAPKs arrests cell cycle and channeling towards pavement cell differentiation [4], [12]. Our results suggest that this is a reversible state, where inactivation of MAPKs through AP2C3 leads to the derepression of cell cycle arrest and thus cell proliferation. Importantly, this cell proliferation directs cells to enter the stomata differentiation pathway. As RBR1-E2F provide a transcriptional regulatory switch important for the transition from proliferation to differentiation [75] and for regulation of the amplifying stem cell division, e.g. in root meristem [54] we tested components of this pathway. Altered E2F, DP and RBR1 protein amounts in AP2C3 overexpression lines and similar change of E2F in the *yda* mutant suggests a connection of RBR1-E2F pathway to stomata developmental signaling. The reduction of E2FC and DPB amounts in AP2C3oe suggests that MAPK signaling may promote dimerization of these proteins to enhance epidermal cell differentiation, with AP2C3 counteracting this role. A repressor role of the E2FC/DPB complex have been suggested [51] and supported by increased E2FC in dark-repressed meristems [50]. Increased protein amounts of E2FB and DPA in AP2C3-overexpressing plants is in agreement that E2FB/DPA strongly promotes cell proliferation and can override the requirement for auxin in cultured cells [53]. Increasing E2FB/DPA protein amounts were observed during promotion of meristem growth upon dark to light transition and were dependent on the activity of genes involved in constitutive photomorphogenesis [50]. Light-influenced stomata density [3] is mediated by gene activities related to constitutive photomorphogenesis, such as COP1 [76]. Correspondingly, overexpression of E2FA/DPA increases stomata density [47]. Interestingly, the level of RBR1 was increased in AP2C3 overexpression lines, however, not surprisingly as high expression of RBR1 in meristematic cells was reported [39], [54]. RBR1 is important in maintaining stem cell proliferation [54], the entry into and the amplification of stem cell division in the stomata lineage [39]. Stomata-expressed target of E2FA-RBR1 transcriptional complex is CDKB1;1, a mitotic cell cycle regulator [40], [77]. FLP is a MYB-related transcription factor regulating meristemoid cell division. In animal cells E2F, RB and MYB-related transcription factor form a complex known as DREAM, which represses both the cell cycle and cell differentiation-related genes [33]. Whether FLP could be part of such a complex, and target CDKB1;1 remains to be investigated. Nevertheless, a change in CDKB1;1 protein forms in AP2C3 overexpression and *yda* lines, which demonstrate similar stomata cluster phenotypes, indicates that not only CDKB1;1 expression but also posttranslational modifications may be involved in this regulation. Interestingly, stomata overproliferation was restricted to the upper part of the hypocotyl in AP2C3 overexpression lines. This may be related to the potential of cells to divide at this region during hypocotyl growth, while in the lower part of the hypocotyl cell growth is exclusively driven by cell expansion accompanied by endoreduplication [45]. ERL1 was suggested to function in proliferating cells, such as leaf primordial cells, and correspondingly, ERL1::GUS was strongly upregulated in the upper part of hypocotyl in AP2C3oe plants. AP2C3 expression in mature stomata may also be related to the maintenance of diploid state and cell proliferation potential of stomata, while the surrounding pavements cells cease to proliferate as soon as they enter endoreduplication. Our observation of cell “tumours” that seemingly originate from stomata may support this assumption. Interestingly, stomatal lineage genes such as CDKB1;1 [77], ER, ERL1 [11], and YODA [12] continue to be expressed in fully differentiated stomata, yet they regulate the earlier stages of stomata developmental pathway.

### Ectopic Cell Proliferation in AP2C3oe lines often Differentiates Stomata without Asymmetric Divisions of Meristemoids

Ectopic cell proliferation in AP2C3oe lines often produced almost isodiametric cells, which eventually differentiated into stomata suggesting that asymmetric cell divisions were not obligatory for stomata initiation. Similar observations in *mpk6*MPK3RNAi, MKK4-MKK5RNAi and *mpk3mpk6*GVG::MPK6 [4] as well as in *scrm-D* seedlings [78] were reported. Studying the expression of markers representing the various stages of stomata development, we have observed that all became upregulated in AP2C3oe lines, suggesting that AP2C3 either may interfere with stomata development at multiple points as was suggested for MAPKs [79], or promote entry into stomata pathway. This suggests that the subsequent steps are linked to the transit of cells through the defined developmental program. Strong deregulation of cell proliferation by AP2C3 overexpression is also indicated by the presence of multiple nuclei in stomata cells of AP2C3oe plants. Observation of large multinucleate cells in Arabidopsis *mkk6/anq1* or *anp2anp3* MAPKKK mutants as well as generation of multinucleate cells during expression of kinase-negative tobacco NQK1 MAPKK suggested MAPK cascades to be essential for correct cytokinesis [80], [81]. Cytokinesis in Arabidopsis is controlled by a pathway that consists of ANP MAPKKKs, MAPKKs HIK and MKK6/ANQ1, with MPK4 being a probable target of MKK6/ANQ [82]. Thus AP2C3 ability to control activity of MPK4 may lead to inactivation of this kinase in AP2C3oe plants resulting in cytokinesis defects.

Activation of MAPKs by stress factors mobilizes plant stress responses and hormone balance including auxin and ethylene [21], [83], [84]. It is predictable that growth and cell cycle related pathways could be blocked by the same stress signaling pathway. Although MPK3 and MPK6 have broad expression, it appears that in stomata this signaling pathway is not only blocking the proliferation, but also switching the differentiation program of these cells. Interestingly, the same stress MAPK signaling module, when linked to a developmental pathway through YODA, can lead to cell cycle repression in response to secreted peptide ligands regulating stomata development [4], [12]. MKK7 and MKK9 were linked both to the inhibition of meristemoid amplifying divisions, and to the promotion of GMC divisions and stomata differentiation [5]. Based on the AP2C3oe phenotype AP2C3 is likely to regulate the former rather than the latter step of MKK7 and MKK9 action during stomata development

It is intriguing how the signaling specificity of different MAPK cascades sharing the same MAPKK-MAPK module can be achieved to define different outcomes. Specific expression, interaction, and subcellular localization of MAPK-inactivating protein phosphatases can play a fundamental role to determine the specificity, as shown here for KIM-containing AP2Cs in Arabidopsis. The diversity within family of PP2C-type phosphatases in plants suggests that these enzymes could be used to channel signaling pathways to specific cellular responses.

## Materials and Methods

### Molecular cloning and vector construction

The gDNAs or cDNAs of AP2C2 (At1g07160), AP2C3/AtPP2C5 (At2g40180) and AP2C4 (At1g67820) were amplified by PCR (primers are listed in Figure S11) from BAC clones (F10K1, T7M7, F12A21; ABRC stock center) or from an Arabidopsis cDNA library, cloned into pGreenII vector downstream of the CaMV 35S promoter and tagged with 9-mer c-Myc epitope, triple HA or sGFP(S65T). AP2C3-G163D was created using a site-directed mutagenesis kit (Stratagene) and tagged with sGFP(S65T). For conditional expression the AP2C3 gDNA was fused with 9-mer c-Myc epitope or YFP and cloned into estradiol-inducible pER8 vector (GenBank ID AF309825). For yeast two hybrid analysis, AP2C2, AP2C3 and AP2C4 cDNAs were cloned into pBD-GAL4cam (Stratagene). MAP kinase constructs for yeast two hybrid and protoplast transfections as well as creation of the ΔANP1 plasmid has been previously described [26], [42]. mRFP1 was used to tag MAPKs for colocalization studies. The ∼2 kb putative promoter regions of AP2C2, AP2C3, and AP2C4 were cloned by PCR using the BAC clones (described above) fused with GUS. For bimolecular fluorescence complementation (BiFC) the gDNAs or cDNAs of AP2C2, AP2C3 and AP2C4 were cloned into pRT100 vector [85] and N-terminally fused with N-terminal domain (ntd) of YFP. Cloning of MAPKs with C-terminal domain (ctd) of YFP for BiFC has been described previously [26]. Cloning of PP2Cs into pGEX-4T-1 for production of recombinant proteins was performed according to [26]. For cloning of chimeric AP2C3/AP2C1 the N-terminal domains of AP2C3 (amino acids 1–135) and AP2C1 (amino acids 1–146) were cloned by PCR and fused with C-terminal domains of AP2C1 (aa 147–396) and AP2C3 (aa 136–390), respectively. For the chimera AP2C3/HAB1 AP2C3ntd (aa 1–135) and HAB1ctd (aa 197–511) were cloned by PCR. Sequences of used primers are included as supplementary information (Figure S 11).

### Induction of AP2C3 expression in estradiol-inducible AP2C3 lines

To induce AP2C3 over-expression in estradiol-inducible AP2C3 lines seedlings were germinated in multiwell dishes in ½ MS and estradiol (Sigma) was added till 5 µM at 3dpg. Wild-type plants treated with estradiol were used as control.

### Yeast two hybrid library screen and interaction assays

The yeast two hybrid screen of an Arabidopsis cDNA library with the pBD-GAL4cam-AP2C3 bait plasmid was performed in the yeast strain PJ-69A as described previously [26]. Yeast two hybrid interaction assays using AP2C1, AP2C2, AP2C3 and AP2C4 in pBD-GAL4cam were performed in PJ-69A strain.

### Plant material, generation of transgenic lines, genetic crosses and stomata ratio calculation

*Arabidopsis thaliana* ecotype Columbia (Col-0) was used as genetic background, transformed using the floral dipping method, and transgenic seeds were selected on kanamycin or hygromycin-containing Murashige-Skoog (MS) plates, or by spraying soil-grown 8 day old seedlings with Basta (Agrovert). T-DNA insertion lines for AP2C3 (SALK_109986), AP2C2 (GABI-Kat_316F11) and AP2C4 (SALK_000296) were analysed by (RT-)PCR using left border (LB) and gene-specific primers followed by sequencing. Southern blotting was performed as described [26] and these lines were crossed to obtain multiple knock-out mutants. Characterization of the AP2C1 T-DNA insertion line has been described previously [26].

8 independent *AP2C3* constitutively over-expressing lines (GFP-, HA- or c-Myc-tagged) were analyzed for stomata phenotype in T2-T6 generations (phenotypes of several hundred plants were investigated). 23 independent AP2C2 over-expressing lines (9 selected) and 43 independent AP2C4 over-expressing lines (5 selected) were analyzed. For promoter-reporter studies 2 independent AP2C2::GUS, 7 independent AP2C3::GUS (2 selected) and 7 independent AP2C4::GUS (2 selected) were analyzed. For inducible *AP2C3* expression with pER8 vector 11 independent lines with c-Myc tagged AP2C3 and 14 independent lines with YFP-tagged AP2C3 were analyzed. For expression of AP2C3-G163D-GFP 17 independent lines were analyzed and for expression of the chimeric proteins 2 independent lines per construct were analyzed.

AP2C3oe lines were crossed with stomatal marker lines (FAMA::GFP, E1728, MUTE::GUS, ERL1::GUS, FLP::GUS-GFP and TMM::TMM-GFP) [11], [15], [16], [43], [46] and selected lines were analyzed.

For stomata ratio [%] calculation, epidermal cells were counted in phosphatase single, double or triple knock out seedlings. SEM images of abaxial side of cotyledons of 5 dpg seedlings were taken by Hitachi TM-1000 and epidermal cells were counted on the images (∼15 seedlings per line and over 100 epidermal cells per image). Stomata ratio was calculated according to the formula: S[%] = Sn/(Sn+En)x100% (Sn – number of stomata, En – number of all other epidermal cells).

### SEM and CLSM microscopy

For confocal laser scanning microscopy (CLSM), 3 to 12 dpg seedlings expressing GFP- or YFP-tagged proteins were observed by Leica TCS microscope (using Ar/Kr laser) using Leica software. For propidium iodide (PI) staining, seedlings were immersed into 10 µg/mL PI solution for 10 min. and rinsed 1–2 min. in distilled water.

For scanning electron microscopy (SEM), cotyledons were flash frozen in liquid nitrogen and observed by Hitachi TM-1000. For gold coating, seedlings were fixed in 2.5% v/v glutaraldehyde (pH 7.1) at room temperature rolling overnight and next day dehydrated through series of 30%, 50%, 70%, 80%, 90% acetone and twice with 100% acetone each 30 min. at room temperature. Samples were immediately put for critical point drying for 1 h, mounted on stubs and coated with gold. Samples were examined by JEOL JSM-6300 (during EMBO workshop on Electron Microscopy and Stereology in Cell Biology, Ceske Budejovice).

### Cultivation of Arabidopsis cell suspension, protoplast preparation, kinase assays and phosphatase assays, histochemical GUS assays

Cultivation of Arabidopsis cell suspension, protoplast isolation, MAPK and PP2C activities studies were performed as described in [86]. *E. coli* produced recombinant GST fusion proteins of phosphatase and MAP kinase were used for *in vitro* dephosphorylation assays.

The cyclin-dependent kinase (CDK) activity assay on histone 1 (HisI) was done as described in [87]. For immunoprecipitation of CDKs 50 µg of total protein extract was incubated with 20 µL of diluted (25%) p13Suc1 agarose conjugate (Upstate Biotechnology, USA). The beads were rolled for 1 h at 4°C and washed 3 times with 1 mL of SucI buffer (50 mM TrisCl pH 7.4, 250 mM NaCl, 5 mM EGTA, 5 mM EDTA, 0.1% v/v Tween 20, 5 mM NaF, 0.1% v/v NP-40, 0.5 mM PMSF) and 1 time with 0.5 mL of kinase buffer (50 mM Tris pH 7.4, 15 mM MgCl_2_, 5 mM EGTA, 1 mM DTT). All the centrifugation steps were done at 1000 rpm for 1 min. in swing-out centrifuge at 4°C. The residual liquid was removed from the beads with a syringe. The beads were immediately mixed with 20 µL of kinase reaction containing 1 µg/µL histone 1 (Sigma), 1 µCi/µl γ^3^ ^2^P-ATP, 10 µM ATP in kinase buffer. The reaction was incubated 30 min. at room temperature. The reaction was terminated by adding SDS loading buffer, heated at 95°C for 3 minutes, centrifuged shortly (∼10 sec) and 15 µL of sample (∼10 µg of histone) per lane was run on a 12.5% SDS-PAA gel. The gel was stained with Coomassie Blue for 10 min., destained with destainer solution (12.5% v/v glacial acetic acid, 10% v/v methanol) for 2 h changing the destainer every 15 min. The gel was dried on Whatman 3MM paper in a vacuum gel-dryer at 80°C for 1 h and exposed to Kodak Biomax MR film [87]. Antibodies used in this study have been already described [50], [53].

Histochemical GUS reporter assays were performed according to Jefferson (Jefferson et al., 1986).

## Supporting Information

Figure S1 **Interaction and localization of PP2Cs with MAPKs.** (A) Interaction of AP2C3 with MPK1, MPK3, MPK4 and MPK6 in protoplasts using bimolecular fluorescence complementation (BiFC). YFPntd-AP2C3 was co-transfected with YFPctd-MAPKs in Arabidopsis suspension culture protoplasts and the reconstituted fluorescence detected. Fluorescence microscopy and differential interference contrast (DIC) images of protoplasts. Bar  = 10 µm. (B) Colocalization of AP2C3 with MPK3, MPK4 and MPK6 in the nucleus of co-transfected Arabidopsis protoplasts: AP2C3-GFP and MAPKs-mRFP1. Bar  = 10 µm. (C) Localization of AP2C2-GFP and AP2C4-GFP in Arabidopsis protoplasts, fluorescence microscopy and differential interference contrast (DIC) images. Bar  = 10 µm.
(TIF)

Figure S2 **Interaction of Arabidopsis PP2Cs with MAPKs in yeast.** (A) Interaction of AP2C1, AP2C2, AP2C3 and AP2C4 with MAPKs in yeast two hybrid assays. Growth of pJ694A yeast cells cotransformed with pBD-PP2C and pAD-MAPKs vectors on selective plates. From top starting clockwise: empty pAD vector, pAD-MPK1, pAD-MPK3, pAD-MPK4, pAD-MPK6. (B) AP2C3 interacts with MPK4 and MPK6. Quantitative β-galactosidase measurements in a yeast two-hybrid assay were performed using pBTM116-AP2C3 in combination with 18 MAPKs in the pGAD424 vector in L40 yeast cells. pBTM116-AP2C1/pGAD424-MPK6 was used as positive control.
(TIF)

Figure S3 **Promoter::GUS staining of AP2C1, AP2C2, AP2C3 and AP2C4 in true leaves 9 dpg. Bar  = 50 µm.**
(TIF)

Figure S4 **Analysis of** ***ap2c3, ap2c2*** **and** ***ap2c4*** **mutant lines.** (A–C) Schematic illustrations of T-DNA insertions and Southern blot analysis of *ap2c2* (GABI-Kat_316F11), *ap2c4* (SALK_000296) and *ap2c3* (SALK_109986) mutant lines. Southern blotting with a labeled LB-specific probe confirmed the presence of single (tandem) T-DNAs within the respective genomes. (D) Detection of *AP2C3* transcript after flagellin (flg22) and cyclohexamide (CHX) treatment in WT and *ap2c3* mutant plants using semi-quantitative RT-PCR. (E) Epidermal surface of *ap2c3* mutant line (bar  = 50 µm).
(TIF)

Figure S5 **Stomata ratio (%) in phosphatase single, double and triple knock-out 3 dpg seedlings.** Epidermal cells were counted in abaxial epidermis of cotyledons of *ap2c1*, *ap2c2*, *ap2c3*, *ap2c4*, *ap2c1ap2c3*, *ap2c2ap2c3*, *ap2c4ap2c3* and *ap2c1ap2c2ap2c3* lines. Stomata ratio was calculated according to the formula: S[%] = Sn/(Sn+En)x100 (Sn – number of stomata, En – number of all other epidermal cells). Error bars indicate standard deviation.
(TIF)

Figure S6 **Analysis of protein expression in AP2C3, AP2C2 and AP2C4 overexpressing lines.** Western analysis of protein expression in independent transgenic plant lines of AP2C3 HA (MW∼42 kDa), and AP2C3-GFP (MW∼70 kDa); AP2C2-GFP (MW∼68 kDa), AP2C2-Myc (MW∼56 kDa) and AP2C2-HA (MW∼45 kDa); AP2C4-GFP (MW ∼76 kDa) and AP2C4-HA (54 kDa), using GFP, Myc or HA antibodies, respectively.
(TIF)

Figure S7 **Estradiol-induced AP2C3 expression.** Immunoblot of protein extracts derived from independent lines transformed with XVE::AP2C3-YFP. GFP antibody was used to detect AP2C3 protein 7 days after application of 5 µM estradiol (+) or without application (−) of independent transformed lines at 10 dpg. Tubulin was detected with tubulin antibody and Ponceau-S staining was used to visualize loading.
(TIF)

Figure S8 **Plant phenotypes.** Comparison of 1-month-old WT and AP2C3oe plants in the soil. AP2C3oe plants show dwarf phenotype.
(TIF)

Figure S9 **AP2C3-overexpression induces stomata marker ERL1.** Upregulation of stomata marker ERL1::GUS in AP2C3oe seedlings. Promoter activity of receptor-like kinase ERL1 is strongly upregulated in 35S::AP2C3-HA monitored at 5 dpg in comparison to WT seedlings after staining for 4 h. Bars  = 50 µm.
(TIF)

Figure S10 ***in vitro*** **phosphatase assay of AP2C3 and AP2C3-G163D proteins.** (A) Schematic representation of AP2C3-G163D mutation. (B) Phosphatase activities of recombinant AP2C3 and AP2C3-G163D proteins towards [32P] phospho-casein monitored by measuring the release of free phosphate. Error bars indicate standard deviation.
(TIF)

Figure S11 **Primers used in this study.**
(TIF)
