# Supplementary figures and images for: MAPK Phosphatase AP2C3 Induces Ectopic Proliferation of Epidermal Cells Leading to Stomata Development in Arabidopsis
Source: PLoS One. 2010 Dec 23;5(12):e15357. doi: 10.1371/journal.pone.0015357 (PMC3009721; doi:10.1371/journal.pone.0015357)

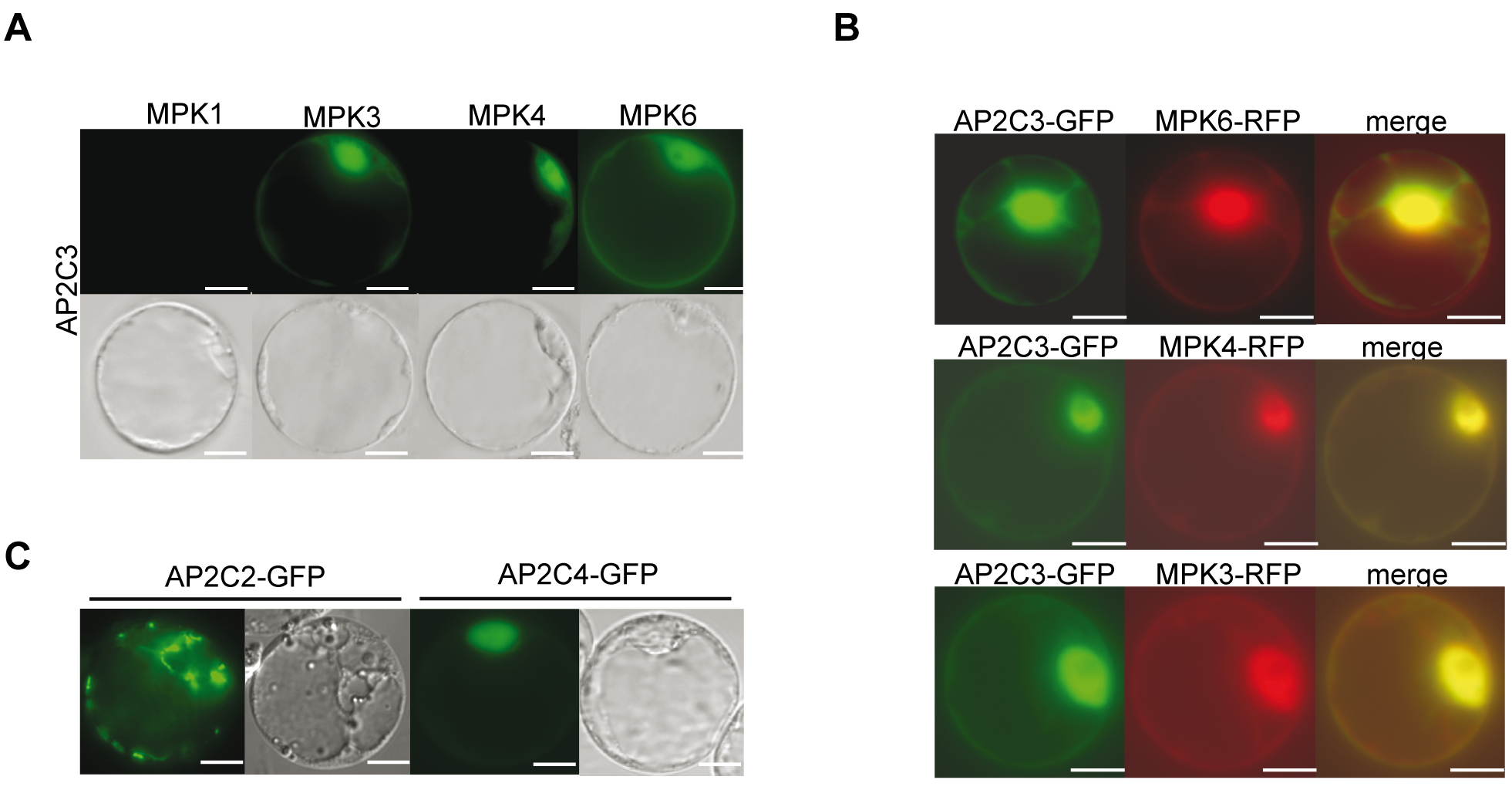

Supplement: Figure S1 — Interaction and localization of PP2Cs with MAPKs. (A) Interaction of AP2C3 with MPK1, MPK3, MPK4 and MPK6 in protoplasts using bimolecular fluorescence complementation (BiFC). YFPntd-AP2C3 was co-transfected with YFPctd-MAPKs in Arabidopsis suspension culture protoplasts and the reconstituted fluorescence detected. Fluorescence microscopy and differential interference contrast (DIC) images of protoplasts. Bar = 10 µm. (B) Colocalization of AP2C3 with MPK3, MPK4 and MPK6 in the nucleus of co-transfected Arabidopsis protoplasts: AP2C3-GFP and MAPKs-mRFP1. Bar = 10 µm. (C) Localization of AP2C2-GFP and AP2C4-GFP in Arabidopsis protoplasts, fluorescence microscopy and differential interference contrast (DIC) images. Bar = 10 µm. (TIF) [file pone.0015357.s001.tif]

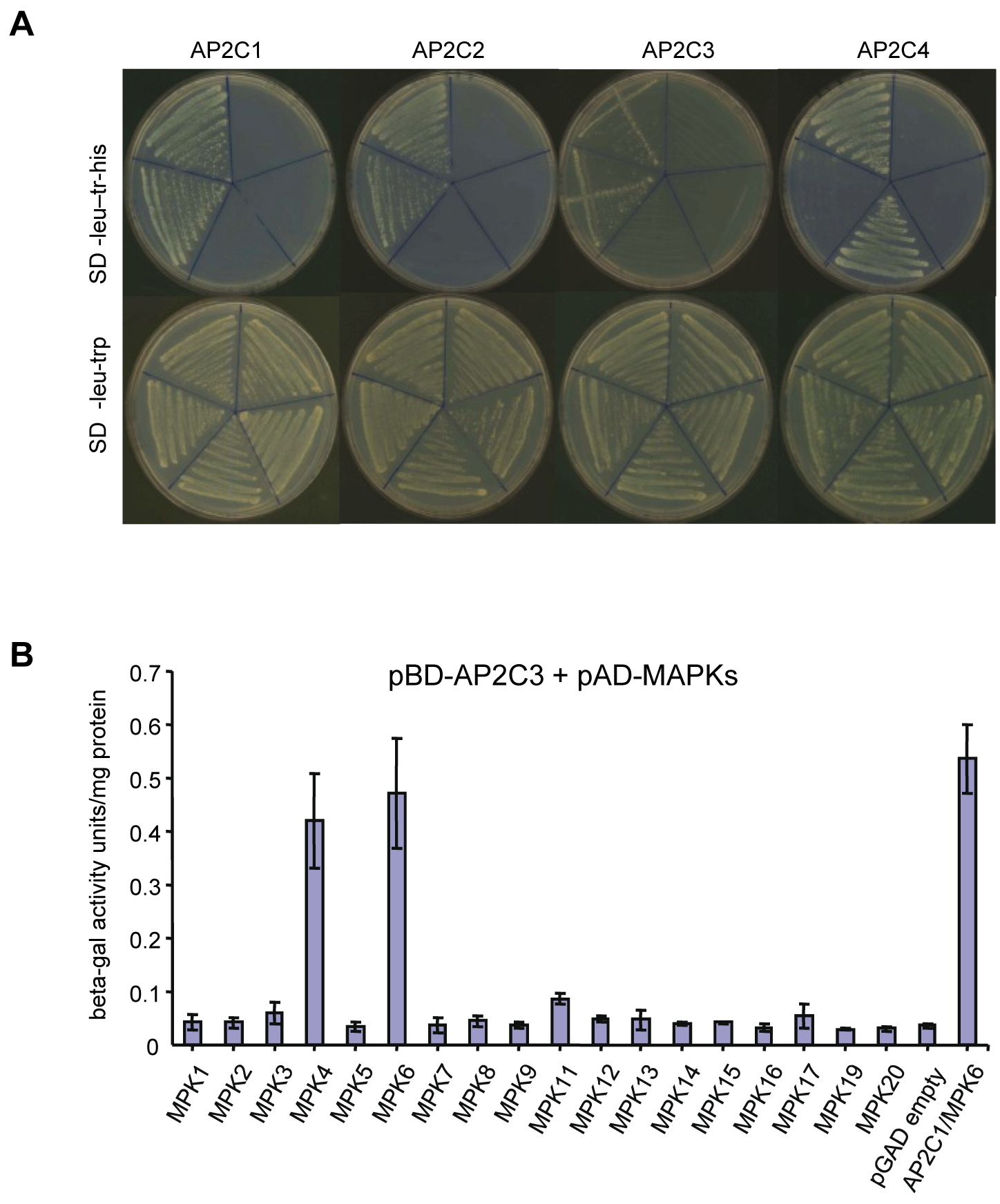

Supplement: Figure S2 — Interaction of Arabidopsis PP2Cs with MAPKs in yeast. (A) Interaction of AP2C1, AP2C2, AP2C3 and AP2C4 with MAPKs in yeast two hybrid assays. Growth of pJ694A yeast cells cotransformed with pBD-PP2C and pAD-MAPKs vectors on selective plates. From top starting clockwise: empty pAD vector, pAD-MPK1, pAD-MPK3, pAD-MPK4, pAD-MPK6. (B) AP2C3 interacts with MPK4 and MPK6. Quantitative β-galactosidase measurements in a yeast two-hybrid assay were performed using pBTM116-AP2C3 in combination with 18 MAPKs in the pGAD424 vector in L40 yeast cells. pBTM116-AP2C1/pGAD424-MPK6 was used as positive control. (TIF) [file pone.0015357.s002.tif]

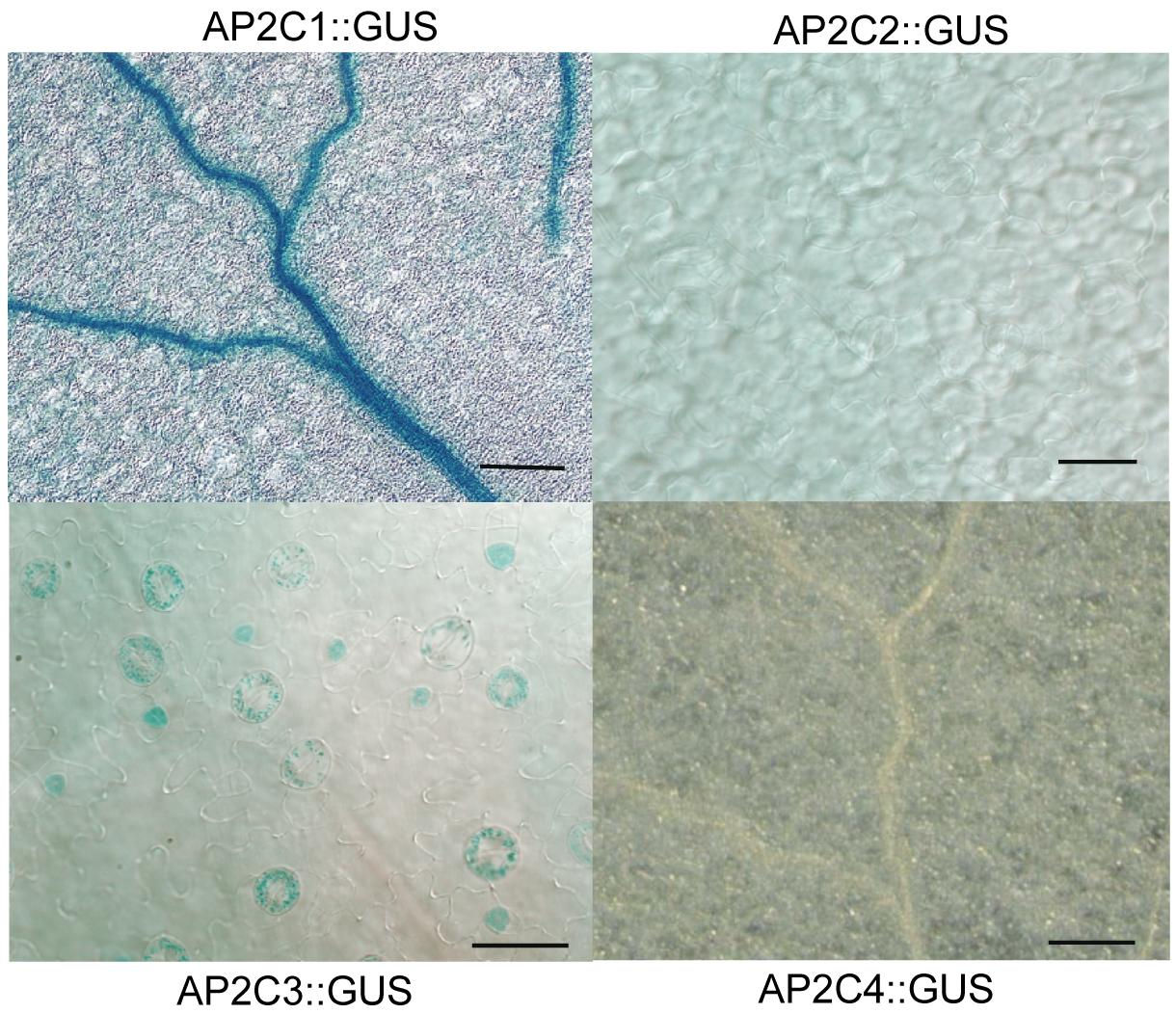

Supplement: Figure S3 — Promoter::GUS staining of AP2C1, AP2C2, AP2C3 and AP2C4 in true leaves 9 dpg. Bar = 50 µm. (TIF) [file pone.0015357.s003.tif]

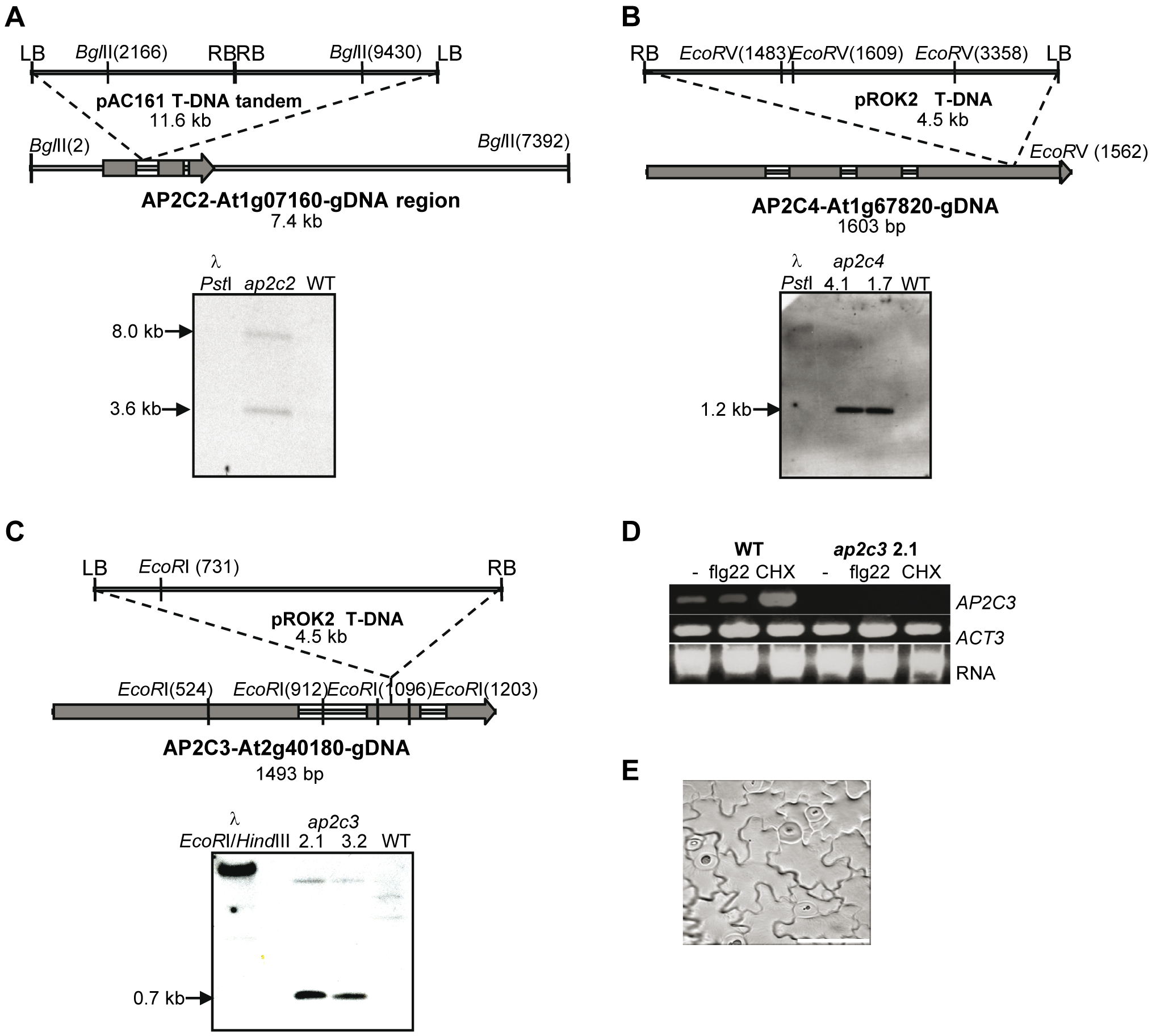

Supplement: Figure S4 — Analysis of ap2c3, ap2c2 and ap2c4 mutant lines. (A–C) Schematic illustrations of T-DNA insertions and Southern blot analysis of ap2c2 (GABI-Kat_316F11), ap2c4 (SALK_000296) and ap2c3 (SALK_109986) mutant lines. Southern blotting with a labeled LB-specific probe confirmed the presence of single (tandem) T-DNAs within the respective genomes. (D) Detection of AP2C3 transcript after flagellin (flg22) and cyclohexamide (CHX) treatment in WT and ap2c3 mutant plants using semi-quantitative RT-PCR. (E) Epidermal surface of ap2c3 mutant line (bar = 50 µm). (TIF) [file pone.0015357.s004.tif]

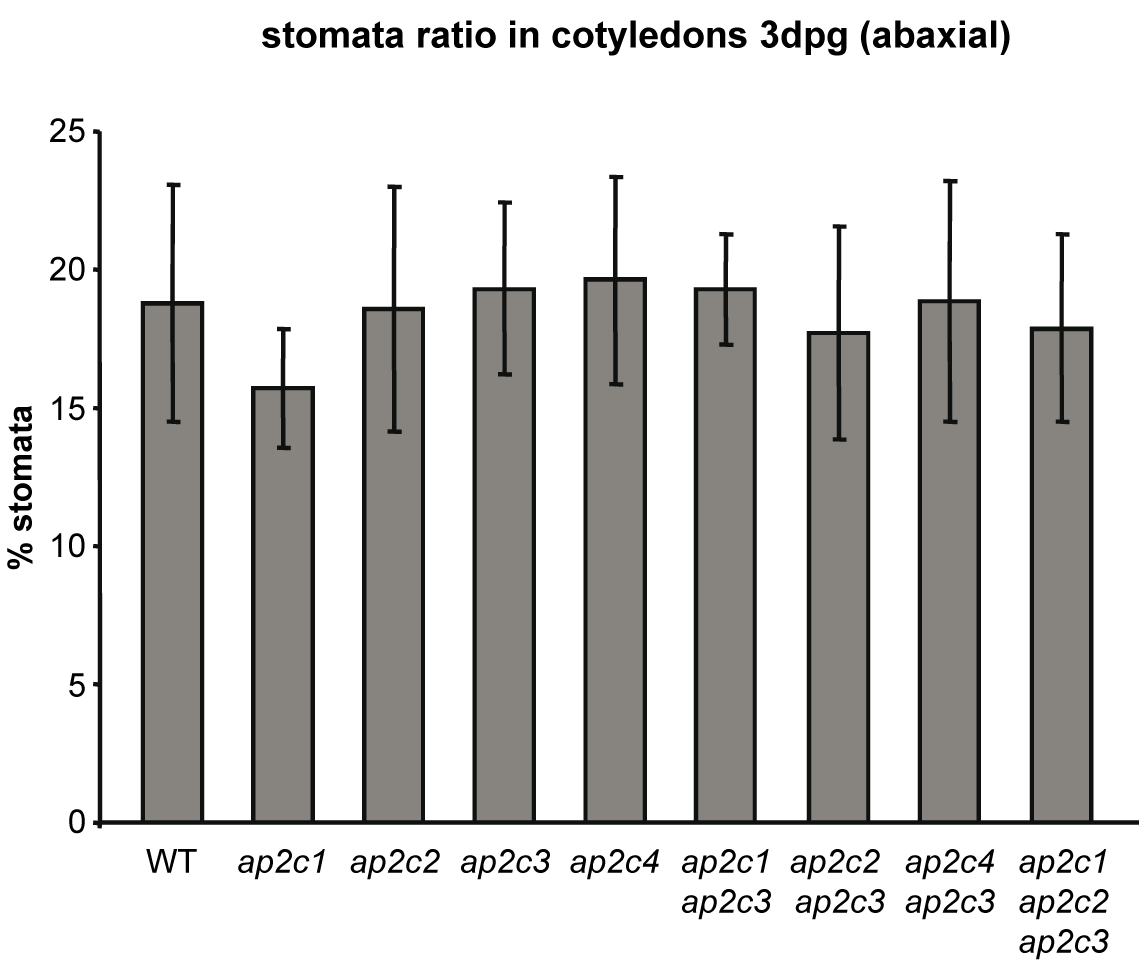

Supplement: Figure S5 — Stomata ratio (%) in phosphatase single, double and triple knock-out 3 dpg seedlings. Epidermal cells were counted in abaxial epidermis of cotyledons of ap2c1, ap2c2, ap2c3, ap2c4, ap2c1ap2c3, ap2c2ap2c3, ap2c4ap2c3 and ap2c1ap2c2ap2c3 lines. Stomata ratio was calculated according to the formula: S[%] = Sn/(Sn+En)x100 (Sn – number of stomata, En – number of all other epidermal cells). Error bars indicate standard deviation. (TIF) [file pone.0015357.s005.tif]

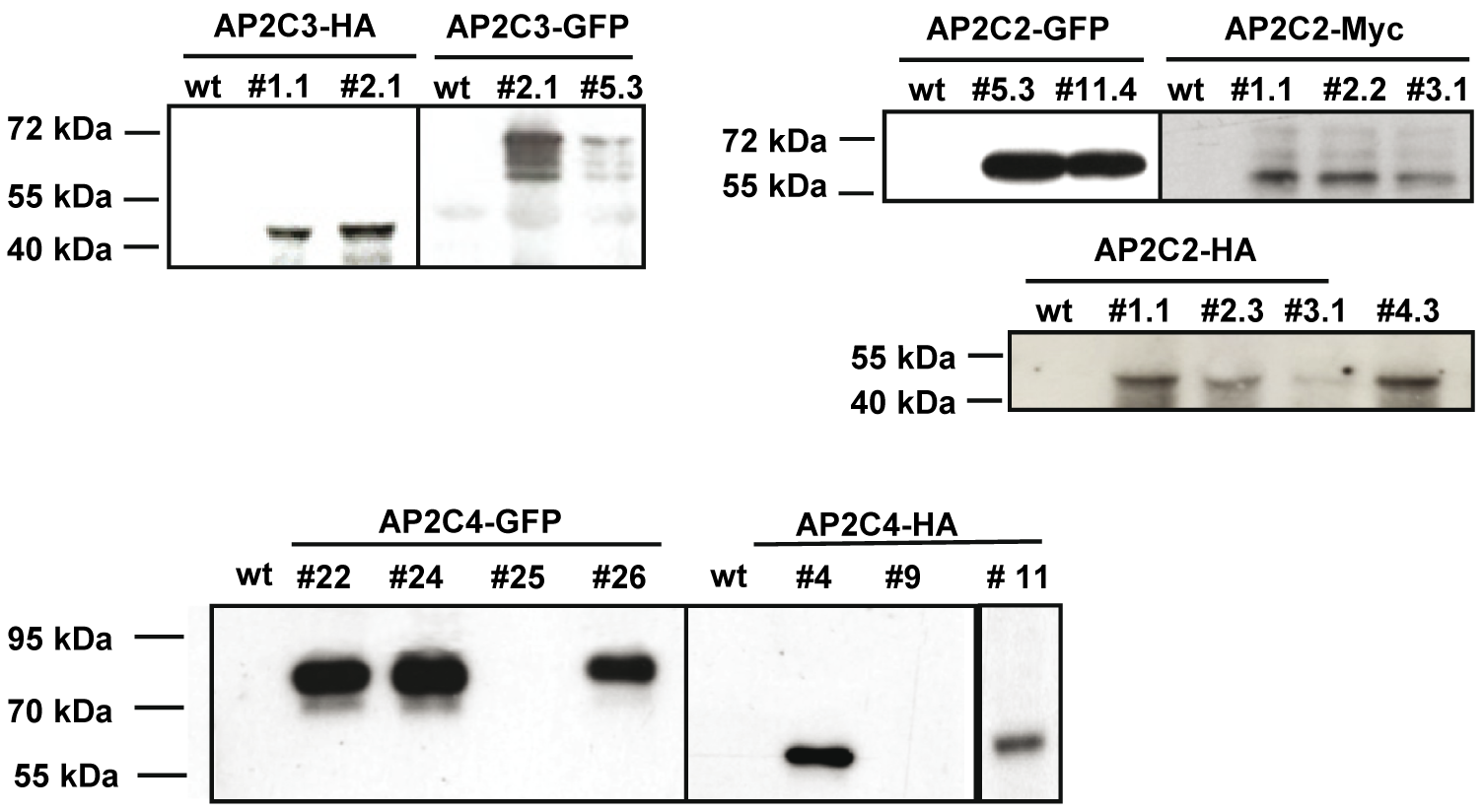

Supplement: Figure S6 — Analysis of protein expression in AP2C3, AP2C2 and AP2C4 overexpressing lines. Western analysis of protein expression in independent transgenic plant lines of AP2C3 HA (MW∼42 kDa), and AP2C3-GFP (MW∼70 kDa); AP2C2-GFP (MW∼68 kDa), AP2C2-Myc (MW∼56 kDa) and AP2C2-HA (MW∼45 kDa); AP2C4-GFP (MW ∼76 kDa) and AP2C4-HA (54 kDa), using GFP, Myc or HA antibodies, respectively. (TIF) [file pone.0015357.s006.tif]

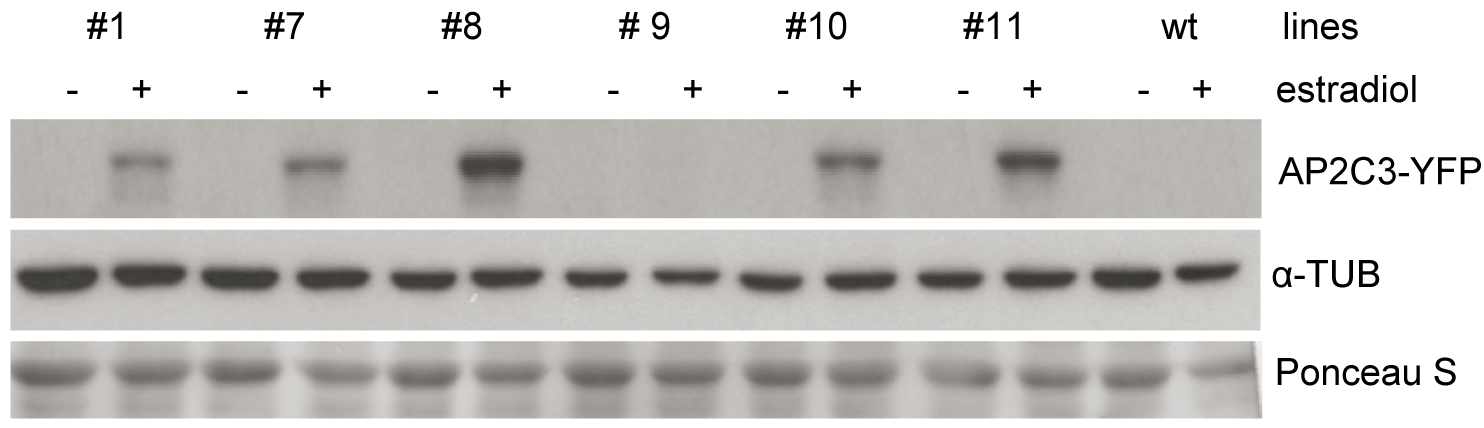

Supplement: Figure S7 — Estradiol-induced AP2C3 expression. Immunoblot of protein extracts derived from independent lines transformed with XVE::AP2C3-YFP. GFP antibody was used to detect AP2C3 protein 7 days after application of 5 µM estradiol (+) or without application (−) of independent transformed lines at 10 dpg. Tubulin was detected with tubulin antibody and Ponceau-S staining was used to visualize loading. (TIF) [file pone.0015357.s007.tif]

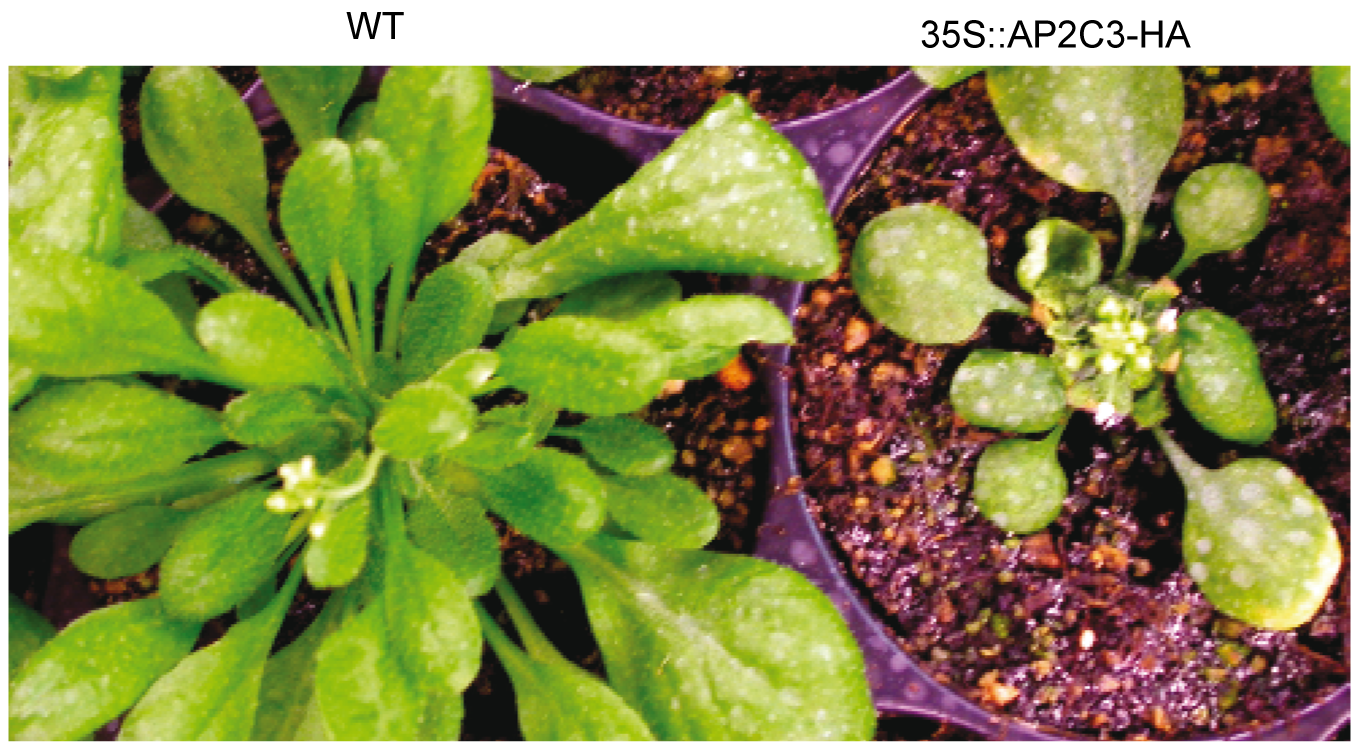

Supplement: Figure S8 — Plant phenotypes. Comparison of 1-month-old WT and AP2C3oe plants in the soil. AP2C3oe plants show dwarf phenotype. (TIF) [file pone.0015357.s008.tif]

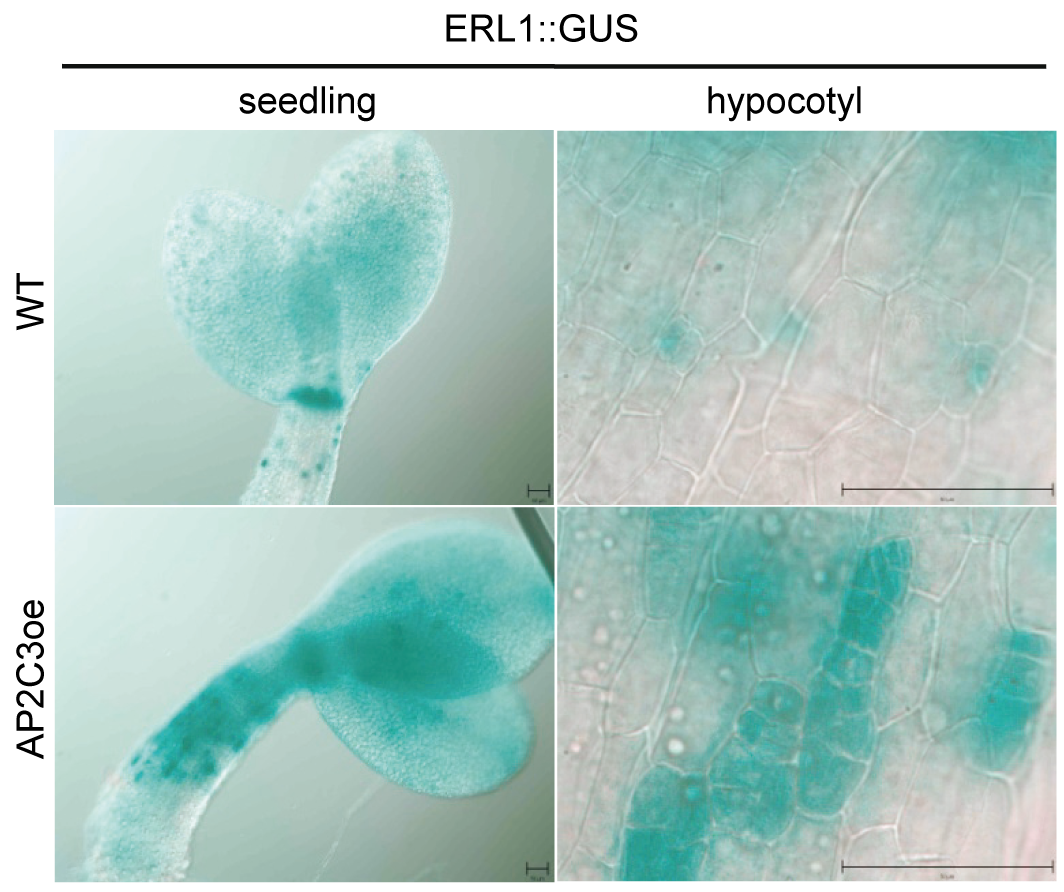

Supplement: Figure S9 — AP2C3-overexpression induces stomata marker ERL1. Upregulation of stomata marker ERL1::GUS in AP2C3oe seedlings. Promoter activity of receptor-like kinase ERL1 is strongly upregulated in 35S::AP2C3-HA monitored at 5 dpg in comparison to WT seedlings after staining for 4 h. Bars = 50 µm. (TIF) [file pone.0015357.s009.tif]

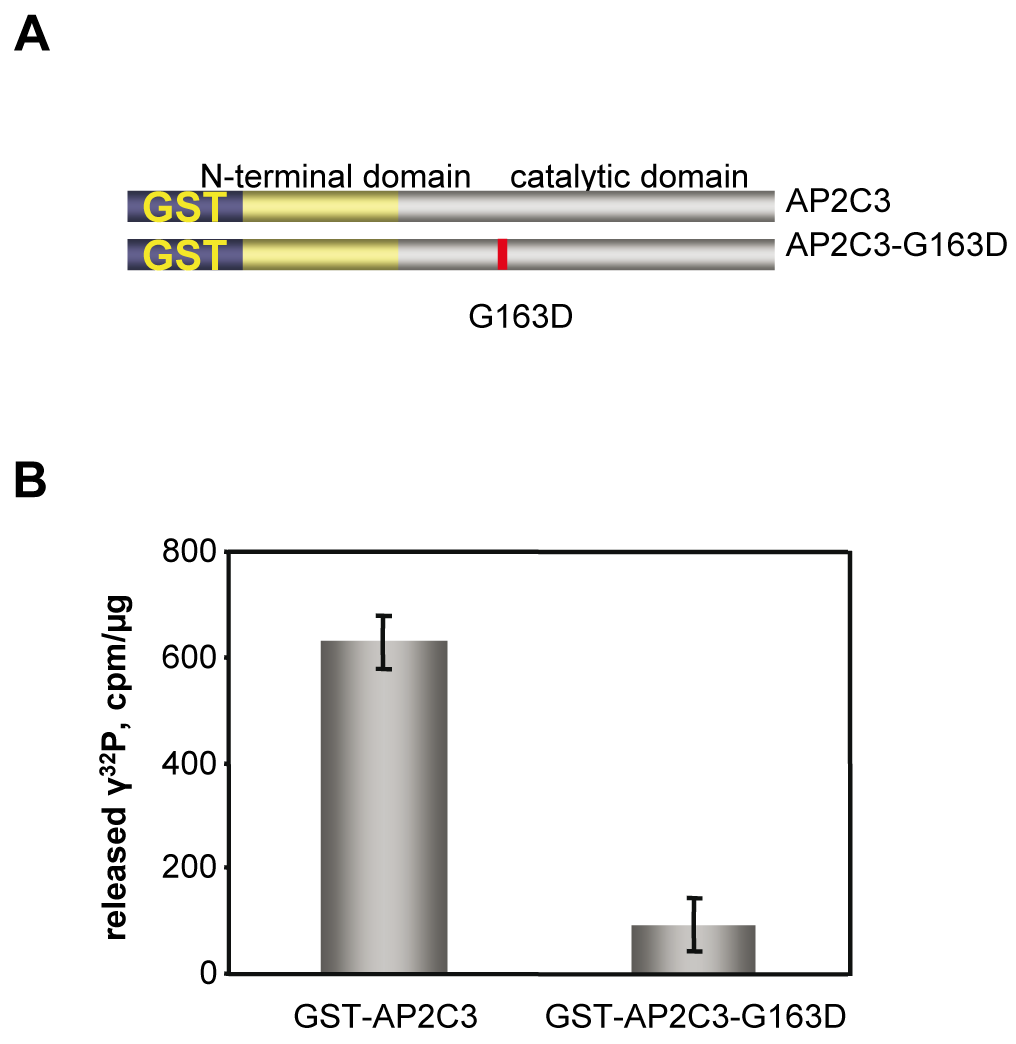

Supplement: Figure S10 — in vitro phosphatase assay of AP2C3 and AP2C3-G163D proteins. (A) Schematic representation of AP2C3-G163D mutation. (B) Phosphatase activities of recombinant AP2C3 and AP2C3-G163D proteins towards [32P] phospho-casein monitored by measuring the release of free phosphate. Error bars indicate standard deviation. (TIF) [file pone.0015357.s010.tif]

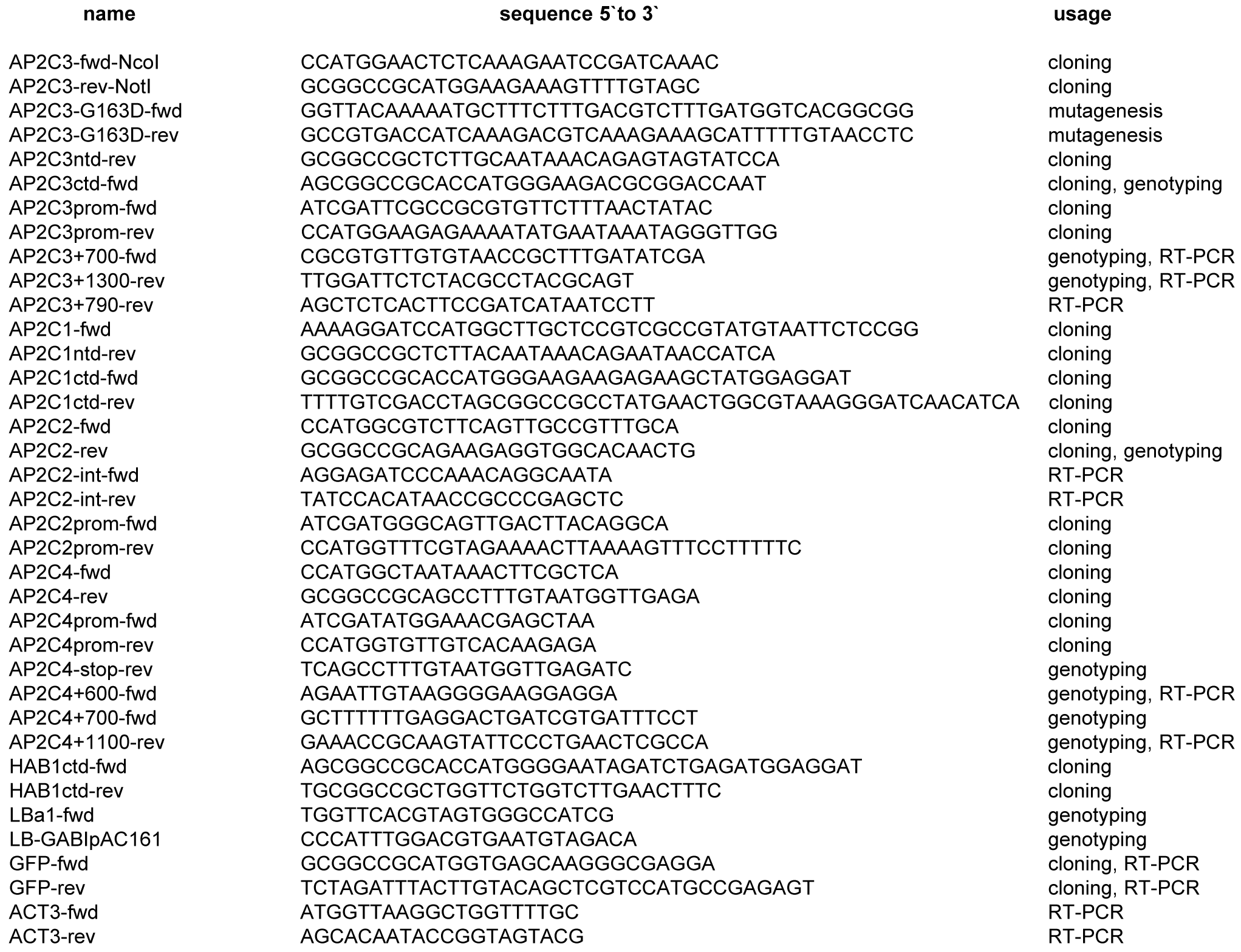

Supplement: Figure S11 — Primers used in this study. (TIF) [file pone.0015357.s011.tif]
